# In silico structural and functional characterization of high-risk missense variants in *MMP8*, *GZMK*, and *OASL* genes associated with epidemic viral infections

**DOI:** 10.1038/s41598-026-40467-w

**Published:** 2026-03-10

**Authors:** Mohamed Et-tanjaouy, Asmae Saih, Omar Machich, Abdelkrim Guendouzi, Younes Zaid, Hanaa Abdelmoumen

**Affiliations:** 1 Laboratory of Microbiology and Molecular Biology, Faculty of Sciences of Rabat, Mohammed V University in Rabat, Rabat, Morocco; 2 Laboratory of Biology and Health, Faculty of Sciences Ben, URAC 34, M’Scik Hassan II University of Casablanca, Casablanca, Morocco; 3 Laboratory of Physiology and Physiopathology, Department of Biology, Faculty of Science, Mohammed V University in Rabat, Rabat, Morocco; 4 Laboratory of Chemistry: Synthesis, Properties and Applications. (LCSPA), Faculty of Sciences, University of Saida - Dr Moulay Tahar, Saida, Algeria; 5 Materials, Nanotechnologies and Environment Laboratory, Department of Biology, Faculty of Sciences, Mohammed V University in Rabat, Rabat, Morocco

**Keywords:** MMP8, GZMK, OASL, bioinformatic tool, SNP, gene, molecular dynamic simulation, Computational biology and bioinformatics, Drug discovery, Structural biology

## Abstract

This research explores the structural and functional consequences of high-impact missense variants in three immune-related genes MMP8 (D253N, Y261S), GZMK (A42P, L122P), and OASL (W216C) with possible relevance to host response in epidemic viral infections. A layered computational workflow was implemented to predict pathogenicity, evaluate structural stability, and assess residue conservation. Subsequent modeling of protein dynamics included structural perturbation analyses, molecular docking, and long-timescale molecular dynamics simulations. Findings revealed that the D253N variant in MMP8 induces substantial deviations from native architecture, characterized by reduced molecular dimensions, lower solvent accessibility, and a broader conformational ensemble. Y261S, by contrast, preserved global folding features with restrained atomic fluctuations. In GZMK, the L122P mutation significantly increased local flexibility and altered compactness, while A42P had minor impact. The W216C substitution in OASL disrupted packing density and expanded surface exposure, indicating a relaxation of the native fold. Principal component analysis confirmed that D253N, L122P, and W216C drive enhanced structural variance relative to native forms. Despite retained ligand-binding capacity, structural rearrangements affected interaction patterns in docking complexes. These findings underscore the potential role of these variants in modifying protein behavior during immune responses. The results serve as a foundation for downstream validation studies on their involvement in infection susceptibility and immune dysregulation.

**Supplementary Information:**

The online version contains supplementary material available at 10.1038/s41598-026-40467-w.

## Introduction

Since the beginning of the 21st century, the world has faced 18 viral epidemics, 6 of which have affected the whole world and 12 of which have been classified as regional epidemics. The viruses that have made history in chronological order include the severe acute respiratory syndrome coronavirus SARS-CoV, the H5N1 influenza virus, the Middle East respiratory syndrome coronavirus MERS-CoV, the H1N1 influenza virus, the Ebola virus, and the severe acute respiratory syndrome coronavirus SARS-CoV-2^1^. The emergence and re-emergence of these viral epidemics always had deleterious effects on public health, as indicated by the number of deaths, as well as having a major, substantial impact on the world’s economies, as in the case of COVID-19^2^. Given this frequency of viral emergence, research aimed at understanding the wide-scale and rapid spread of viral infection has pointed to a real need to explore the details of the interaction between the host and the viruses in question. This exploration can reveal critical insights into the mechanisms of viral transmission, potential mutations, and the host’s immune responses. By fostering a more profound understanding of these dynamics, scientists can develop more effective vaccines and therapeutic strategies to mitigate the impact of future outbreaks^3^. To elucidate the details of the host’s response to a viral infection, transcriptomic analysis is the best approach for discovering the key genes involved in this interaction^4^. In our earlier research, we looked at how the host’s response to SARS-CoV-2 infection compared to responses to other viruses like SARS-CoV, MERS-CoV, EBOV, H5N1, and H1N1. Our first findings have highlighted the significance of ten key common host response genes, among them three that we found relevant to prioritize: MMP8, GZMK, and OASL^5^.

The MMP8, or matrix metalloproteinase-8 gene codes for the production of neutrophil collagenase. This enzyme, specifically designed for the degradation of the extracellular matrix, performs essential functions such as tissue remodeling, induction of an antiviral cellular response, regulation of the inflammatory process, and modulation of the immune response. Its function in regulating inflammation by limiting excessive inflammatory responses was confirmed in a study conducted on therapeutic approaches aimed at treating pathological pulmonary fibrosis induced by drugs or respiratory viral infections^6^. In addition, the role of the MMP-8 gene in tissue remodeling has been demonstrated by other studies indicating a significant delay in the healing of skin wounds in collagenase-2 (MMP-8)-deficient mice. These mice showed other valuable signs, such as an altered inflammatory process and slowed neutrophil infiltration^7^. Another significant feature of the MMP-8 gene has been revealed by research on the molecular basis of the immunopathology of SARS-CoV-2, which has revealed its key function in several pathways of interaction between the host and the viral infection. These studies have explored the correlations of MMP-8 expression with the production of bioactive mediators stimulating the antiviral cellular response, as well as affirming its implications in viral immunosuppressive actions^8^. The multifaceted role of the MMP-8 gene in the context of SARS-CoV-2 immunopathology underscores its significance in understanding host-viral interactions. By facilitating the production of bioactive mediators, MMP-8 not only enhances antiviral cellular responses but also highlights a complex interplay that can influence disease outcomes^9^. Moreover, its involvement in viral immunosuppressive actions raises important questions about therapeutic strategies and potential interventions. As research continues to unveil the intricate mechanisms at play, targeting MMP-8 may emerge as a promising avenue for modulating immune responses and improving patient outcomes in the face of viral infections like COVID-19^10^.

The GZMK gene helps make granzyme K, which is a type of enzyme that plays many roles, particularly in adjusting the body’s immune response to infections and fighting viruses. Several studies have shown that memory T cells secrete high levels of granzyme K following viral infection. This secretion is crucial for the elimination of infected cells and the establishment of long-term immunity^11^. The intricate relationship between the GZMK gene and granzyme K underscores a vital aspect of our immune system’s functionality. By facilitating the production of granzyme K, the GZMK gene plays a pivotal role in modulating the immune response, particularly during infections and viral challenges. Memory T cells, renowned for their ability to recall past pathogens, are instrumental in secreting elevated levels of granzyme K following an infection. This secretion is not merely a response but a crucial mechanism for effectively eliminating infected cells and establishing long-lasting immunity^12^. Thus, understanding the dynamics of granzyme K production and its implications paves the way for advancements in immunology and potential therapeutic strategies against infectious diseases. Furthermore, ongoing research is exploring how variations in the GZMK gene could impact immune responses in different individuals. These high levels of granzyme K have been confirmed in people infected with human cytomegalovirus and influenza viruses and in people suffering from viral pneumonia^13^. Other studies have reported an increase in the frequency of cytotoxic T lymphocytes and NK cells expressing this protein in HIV-1 patients. These studies say that granzyme K is crucial for the body’s response to viral infections because it can cause infected cells to die and can also help stop the virus from making more copies of itself. Granzyme K also helps control the immune response by interacting with cytokines and receptors on immune cells, which affects inflammation^14^.

The 2’, 5’-oligoadenylate synthase-like (OASL) gene makes the OASL protein, which plays a big role in helping the body fight off infections. The OASL protein is considered an important part of the body’s defense against viruses, working in different ways, such as breaking down viral and cell RNA with RNase L, stopping virus reproduction, triggering cell death, boosting the body’s natural immune response, and being controlled by cytokines^15^. Host mechanisms against viral infections have been the subject of several studies. One of these studies, which targeted the role and mechanisms of OASL against double-stranded RNA viruses, claimed that its overexpression inhibits replication of the IBDV. This study also demonstrated the enzymatic role of OASL independently of its antiviral activity by targeting the viral protein VP2 to degrade it via the autophagy pathway^15^. Another study on how OASL helps the immune system found that changes in the host cell’s DNA during an IAV infection lead to more OASL protein being made. The promotion of high levels of OASL protein enhances the activation of antiviral interferons via receptors that recognize the patterns of viral infection^16^.

Computational methods, including molecular docking, molecular dynamics (MD) simulations, and structure-based modeling, are increasingly used to investigate host–virus interactions and guide therapeutic development. Agrahari et al. (2023) demonstrated that the SARS-CoV-2 envelope protein employs a lysine acetylation-mediated interaction network to mimic histones and interact with the host protein BRD4, emphasizing the role of post-translational modifications and conformational dynamics^17^. In parallel, structure-guided strategies have been applied to target PPIs. Agrahari et al. (2023) identified ASK1 regulators using crystallographic mining and docking^18^, while Haider et al. (2023) reported an ASK1 inhibitor targeting its catalytic region through a computer-guided screening approach^19^. The purpose of this study is to examine the structural and functional consequences of high-risk missense variants in the human MMP8, GZMK, and OASL genes using in silico approaches. Additionally, it seeks to investigate the potential correlation of these variants with the pathogenesis or host response mechanisms related to epidemic viral infections. By employing computer-based prediction tools and structural analyses, the research intends to identify harmful mutations that could alter protein function, disrupt the immune system, or increase susceptibility to viral diseases such as COVID-19, as well as other outbreaks including MERS-CoV, EBOV, H5N1, and H1N1.

## Materials and methods

### Retrieval of variant datasets

Missense variants of the MMP8, GZMK, and OASL genes were retrieved from the *Ensembl* database^20^. The amino acid sequence of MMP8, GZMK, and OASL were obtained from Uniprot knowledgebase^21^(UniProtKB) (Accession Numbers: P22894, P49863, and Q15646 respectively). Six various pathogenicity predicting tools SIFT^22^, PolyPhen-2^23^, CADD^24^, AlphaMissense^25^, MetaLR^26^, and Mutation Assessor^27^ were utilized to predict the predict the pathogenicity of these nsSNPs. A total of 7,177 SNPs, of which 558 were missense variants, were identified in the MMP8 gene. Of the 4639 SNPs identified in the GZMK gene, 281 missense variants were discovered. The OASL gene included 1211 missense variants among 27,273 SNPs. Only nsSNPs projected to be deleterious by all six tools were selected for further analysis.

### Analyzing the effect on proteins stability

The stability of the tested proteins was checked using four publicly available bioinformatics tools: I. Mutant 3.0^28^, MuPro^29^, DDMut^30^, and INPS-MD^31^. These software tools predict the impact of mutations at a single site on the stabilization of proteins. A score of 4 was allocated according to the number of tools that demonstrated each SNP’s impact on the stability of the selected genes, classified as destabilizing (decrease) or stabilizing (increase, neutral). Every tool that yielded a “destabilizing result” added a “+1 score” to the SNP, whereas each stabilizing result resulted in a “0 score.”

### Evolutionary conservation analysis of MMP8, GZMK, and OASL

Conservation prediction of the MMP8, GZMK, and OASL protein sequences was analyzed with DeepREx-WS^32^. The FASTA sequences of the three selected proteins were provided as the input to ascertain the evolutionary conservation of the predicted deleterious nsSNPs.

### Determining the protein stability, flexibility, and interatomic interactions

We looked at how point mutations affected the stability and flexibility of the MMP8, GZMK, and OASL proteins that controlled how atoms interacted with each other using DynaMut^33^. We supplied the mutation list and WT structure in PDB format as input. With the help of normal mode analysis (NMA) and an elastic network contact model (ENCoM), DynaMut can predict the structure of mCSM^34^, SDM^35^, DUET^36^., and ΔΔG^37^.

### Prediction and validation of 3D structures

The three-dimensional structure was forecasted using an artificial intelligence system, AlphaFold^38^, capable of computationally predicting protein structures with precision and rapidity. The UniProt IDs of the chosen proteins (MMP8, GZMK, and OASL) were utilized as input to obtain the AlphaFold model. Additionally, the selected high-risk missense variations were manually included in the resulting WT structures, utilizing the mutagenesis wizard in PyMoL^39^ to generate the crystallographic models of the identified deleterious SNPs. We performed the conformational validation of the modeled structures for both native and mutant variants using the web-based programs ERRAT^40^, Verify3D^41^, and PROCHECK^42^.

### Normal mode analysis

Normal mode analysis was performed using the iMod server (iMODS)^43^, employing the standard default settings for all specified parameters. This investigation focused solely on mutations identified as highly pathogenic. This methodology facilitated a targeted evaluation of the structural effects these mutations have on protein stability and function.

### Molecular docking analysis

Protein–ligand interactions were evaluated with CB-Dock2, a docking pipeline that couples automated cavity detection, homologous-template fitting, and docking via AutoDock Vina 1.1.2. Blind docking was used to avoid pocket-selection bias across WT and high-risk missense variants of MMP8, GZMK, and OASL, allowing CB-Dock2 to center adaptive grids on the highest-ranked cavities. Ligand structures—doxycycline anhydrous (PubChem CID 54671203), bosutinib (CID 5328940), and astilbin (CID 119258)—were retrieved from PubChem, desalted and protonated at ~ pH 7.4, then converted from SDF to PDB with Open Babel prior to docking. The set was chosen to span complementary chemotypes under an identical docking/MD workflow: doxycycline, a polar Zn-chelating tetracycline, is suitable for metalloprotein environments (e.g., the Zn²⁺ catalytic setting of MMP8); bosutinib, a drug-like heteroaromatic kinase inhibitor, engages deep/hydrophobic pockets through hinge-region and structured-water interactions (useful around GZMK subsites); and astilbin, a bulky polyphenolic glycoside, probes π-rich, surface-exposed regions consistent with OASL recognition surfaces. Docking used rigid receptors and flexible ligands; binding propensity was ranked by the Vina score (kcal/mol), with more negative values indicating stronger predicted affinity. Top poses were then examined and validated by molecular dynamics.

The proteins MMP8, GZMK, and OASL were docked with the ligands doxycycline anhydrous, bosutinib, and astilbin, respectively, using the AutoDock tool from the CB-Dock2 server^44^. This study utilized the PDB files of both the native and mutant protein models as receptor input files. Each ligand was uploaded to the web server in PDB format. The binding affinity of the complexes was evaluated using the Vina score^45^(kcal/mol), where a lower score indicates a higher predicted binding affinity. The stability of the docked complexes was further validated through molecular dynamics simulations^46^.

### Molecular dynamic simulations

To investigate the conformational dynamics and stability of the WT and mutant proteins (MMP8, GZMK, and OASL) in complex with their respective ligands (Doxycycline Anhydrous, Bosutinib, and Astilbin), we performed all-atom molecular dynamics (MD) simulations using the GROMACS-23-GPU package^47^. The CHARMM36-2022 force field^48^ was applied to the protein atoms, and ligand topologies were generated using the SwissParam server^49^. Each protein–ligand complex was solvated in a dodecahedral box with a 1.0 nm minimum distance to the box edge, using the TIP3P water model^50^. Systems were neutralized with Na⁺ or Cl⁻ ions as needed and subsequently energy-minimized using the steepest descent algorithm until the maximum force (Fmax) fell below 1000 kJ/mol·nm.

The systems were then equilibrated in two phases: first, under the NVT ensemble for 1 ns at 310.5 K using the V-rescale thermostat (τ_t = 0.1 ps), followed by the NPT ensemble for 1 ns at the same temperature and a pressure of 1 bar, maintained by the Parrinello–Rahman barostat (τ_p = 2.0 ps). Production MD simulations were run for 250 ns for each system. To ensure statistical robustness, three independent replicates were performed for each complex, starting from different initial velocities. The production runs used an integration timestep of 2 fs, with all bonds involving hydrogen atoms constrained using the LINCS algorithm. Non-bonded interactions were treated with a cutoff of 1.2 nm for both van der Waals and short-range electrostatic interactions, while long-range electrostatics were handled using the Particle Mesh Ewald (PME) method with a Fourier spacing of 0.16 nm. The neighbor list was updated every 20 steps, and trajectory coordinates and energies were saved every 10 ps for subsequent analysis.

The stability and dynamic behavior of each system were assessed by calculating the root mean square deviation (RMSD), root mean square fluctuation (RMSF), radius of gyration (Rg), and solvent accessible surface area (SASA). Principal component analysis (PCA)^51^ was also performed to characterize essential collective motions. All analyses were conducted using built-in GROMACS tools and were averaged over the three independent replicates.

In order to estimate the binding free energy between the WT and mutant complexes of MMP8, GZMK, and OASL with their respective ligands (doxycycline, bosutinib, and astilbin), MM/PBSA analysis was performed on the last 20 ns of the molecular dynamics trajectories using the g_mmpbsa package^52^. The reported MM/PBSA binding free energies were obtained from a single continuous molecular dynamics trajectory and correspond to averages over multiple snapshots rather than single-frame values. MM/PBSA calculations were performed using frames extracted from the production trajectory between frame 6073 and frame 24,934, with an interval of 10 frames, resulting in a total of 1,887 snapshots used in the analysis. The binding free energy was decomposed into van der Waals energy (ΔE_vdW), electrostatic energy (ΔE_ele), polar solvation energy (ΔG_polar), and nonpolar solvation energy (ΔG_nonpolar). The total binding free energy (ΔG_total) and the contribution of each energy component for all studied complexes are reported in the corresponding tables.

## Results

### Computational assessment of deleterious high-risk missense variants

A full set of computer programs was used to examine missense variants in the MMP8, GZMK, and OASL genes to see how they could impact function and structure. SIFT, PolyPhen 2, MetaLR, and Mutation Assessor all consistently identified the MMP8 changes D253N and Y261S as harmful or possibly deleterious, with CADD scores of 31 and 32, respectively, and AlphaMissense values of 0.927 and 0.848. SIFT, PolyPhen 2, MetaLR, and Mutation Assessor all agreed that the changes in the MMP8 gene, D253N and Y261S, could be deleterious or possibly damaging, with CADD scores of 31 and 32 and AlphaMissense values of 0.927 and 0.848. SIFT, PolyPhen 2, MetaLR, and Mutation Assessor all agreed that the changes in the MMP8 gene, D253N and Y261S, could be harmful or possibly damaging, with CADD scores of 31 and 32 and AlphaMissense values of 0.927 and 0.848. Scores of 0.667 and 0.802 for MutPred2 indicated possible-to-probable pathogenicity. I- Mutant 3.0, MuPro, DDMut, and INPS MD all anticipated a decrease in stability and a destabilizing effect. SIFT, PolyPhen 2, AlphaMissense, MetaLR, and Mutation Assessor all showed that GZMK A42P and L122P are deleterious. Scores above 0.9 for MutPred2 validated their functional importance even more. I-Mutant, MuPro, DDMut, and INPS-MD predict structural instability, with L122P having very high scores. The W216C mutation in OASL showed a strong disease-causing potential, receiving the highest scores from PolyPhen 2 and AlphaMissense and was predicted to cause instability by MuPro, DDMut, and INPS MD. DeepREx WS found that all the changed parts in the three genes are mostly similar across different species and likely hidden inside, which means these changes could affect the basic structure or function of the proteins. When taken together, these results support the hypothesis that the studied polymorphisms could be physiologically significant and impair protein function or integrity. Tables 1, 2, and 3 present the predicted effects of nsSNPs in the MMP8, GZMK, and OASL genes, as evaluated using various computational tools.

**Table 1:** Possible prediction results of missense variants using software tools in the MMP8 gene.

SNP number	rs1294178354	rs776868089
Amino acid change	D253N	Y261S
SIFT	0.00 (Deleterious)	0.00 (Deleterious)
PolyPhen 2.0	1 (Probably damaging)	1.000 (Probably damaging)
AlphaMissense	0.927 (Pathogenic)	0.848 (Pathogenic)
CADD	31 (Harmful)	32 (Harmful)
MetaLR	0.582 (Deleterious)	0.645 (Deleterious)
Mutation Assessor	0.984 (Deleterious)	0.994 (Deleterious)
I.Mutant 3.0	8 (Decrease)	7 (Decrease)
MuPro	− 0.96947633 (Decrease)	− 1.4613113 (Decrease)
DDMut	− 1.44 (Destabilizing)	− 2.78 (Destabilizing)
INPS-MD	− 0.62 (Weakly destabilizing)	− 1.85 (Destabilizing)
DeepREx-WS	0.3614 (Highly conserved and buried)	0.38531 (Highly conserved and buried)

**Table 2:** Possible prediction results of missense variants using software tools in the GZMK gene.

SNP number	rs747694136	rs781500556
Amino acid change	A42P	L122P
SIFT	0.00 (Deleterious)	0.00 (Deleterious)
PolyPhen 2.0	0.965 (Probably damaging)	1.000 (Probably damaging)
AlphaMissense	0.934 (Pathogenic)	0.951 (Pathogenic)
CADD	31 (Harmful)	31 (Harmful)
MetaLR	0.887 (Deleterious)	0.921 (Deleterious)
Mutation Assessor	0.926 (Deleterious)	0.954 (Deleterious)
I.Mutant 3.0	7 (Decrease)	7 (Decrease)
MuPro	− 1.1429308 (Decrease)	− 2.1174707 (Decrease)
DDMut	− 0.38 (Destabilizing)	− 3.25 (Destabilizing)
INPS-MD	− 1.83 (Destabilizing)	− 3.19 (Destabilizing)
DeepREx-WS	0.20068 (Highly conserved and buried)	0.33454 (Highly conserved and buried)

**Table 3:** Possible prediction results of missense variant using software tools in the OASL gene.

SNP number	rs757850225
Amino acid change	W216C
SIFT	0.00 (Deleterious)
PolyPhen 2.0	1.000 (Probably damaging)
AlphaMissense	0.968 (Pathogenic)
CADD	30 (Harmful)
MetaLR	0.716 (Deleterious)
Mutation Assessor	0.914 (Deleterious)
I.Mutant 3.0	8 (Decrease)
MuPro	− 0.60030702 (Decrease)
DDMut	− 1.56 (Destabilizing)
INPS-MD	− 1.48 (Destabilizing)
DeepREx-WS	0.26847 (Highly conserved and buried)

**Table 4:** Predicted stability changes (ΔΔG) and molecular flexibility variations for selected amino acid substitutions in the MMP8, GZMK, and OASL proteins using DynaMut.

Protein	Amino acid variant	ΔΔG DynaMut (kcal/mol)	ΔΔG ENCoM (kcal/mol)	ΔΔG mCSM (kcal/mol)	ΔΔG SDM (kcal/mol)	ΔΔG DUET (kcal/mol)	ΔΔS_vib_ ENCoM (kcal mol^−1^ K^−1^)
MMP8	D253N	− 1.042 (Destabilizing)	− 0.151 (Destabilizing)	− 1.137 (Destabilizing)	− 0.670 (Destabilizing)	− 0.996 (Destabilizing)	0.189 (Increase of molecule flexibility)
	Y261S	− 2.964 (Destabilizing)	− 1.088 (Destabilizing)	− 3.163 (Destabilizing)	− 3.210 (Destabilizing)	− 3.377 (Destabilizing)	1.360 (Increase of molecule flexibility)
GZMK	A42P	− 0.286 (Destabilizing)	− 0.097 (Destabilizing)	− 0.154 (Destabilizing)	− 4.130 (Destabilizing)	− 0.751 (Destabilizing)	− 0.121 (Decrease of molecule flexibility)
	L122P	− 2.884 (Destabilizing)	− 0.351 (Stabilizing)	− 2.241 (Destabilizing)	− 4.310 (Destabilizing)	− 2.944 (Destabilizing)	0.439 (Increase of molecule flexibility)
OASL	W216C	− 0.516 (Destabilizing)	− 0.627 (Destabilizing)	− 1.279 (Destabilizing)	− 1.710 (Destabilizing)	− 1.158 (Destabilizing)	0.783 (Increase of molecule flexibility)

**Fig. 1:**
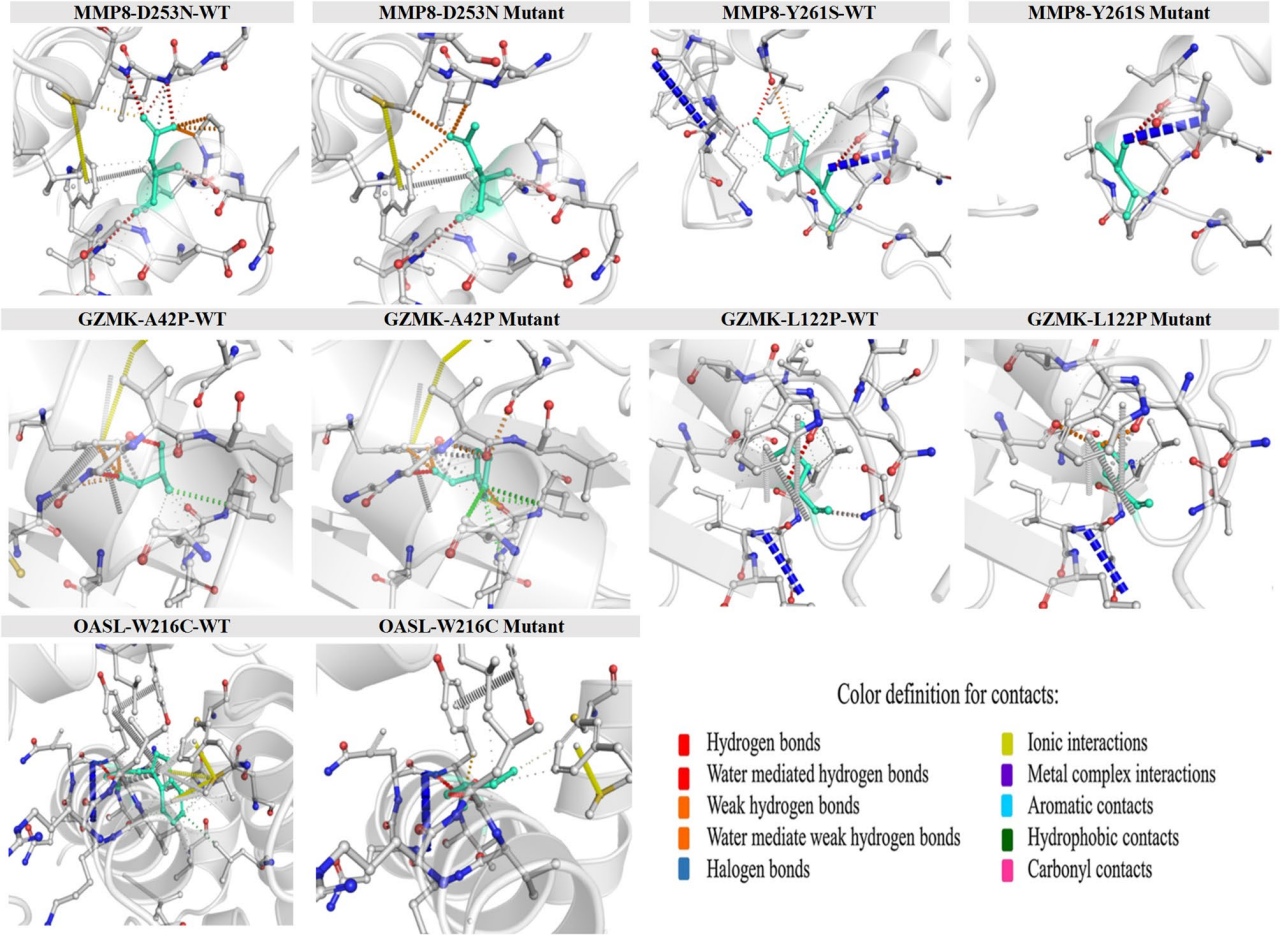
Structural visualization of WT and mutant forms of MMP8 (D253N, Y261S), GZMK (A42P, L122P), and OASL (W216C) proteins. The 3D models illustrate key residue interactions before and after mutation, highlighting changes in hydrogen bonding, hydrophobic contacts, and other molecular interactions. Visualizations were generated to compare residue environments and contact networks using molecular interaction mapping.

The DynaMut server was utilized to assess the predicted interatomic interactions of five high-risk missense variants selected from previous analyses. It provided predictions regarding the changes in energy and vibrational entropy between the mutant proteins and their normal counterparts. Structural analysis conducted with the DynaMut tool indicated a generally destabilizing effect of the selected amino acid substitutions in the MMP8, GZMK, and OASL proteins. In MMP8, the Y261S and D253N mutations displayed ΔΔG values of -2.964 and − 1.042 kcal/mol, respectively, suggesting a significant loss of structural stability, particularly for the Y261S mutation. In the GZMK protein, the L122P mutation exhibited a strong destabilizing effect (-2.884 kcal/mol), while the A42P mutation had a milder impact (-0.286 kcal/mol). Additionally, the OASL W216C mutation resulted in noticeable destabilization, with a ΔΔG of -0.516 kcal/mol (Table 4). Figure 1 illustrates the differences in interatomic interactions, including hydrogen bonds and ionic interactions, between the WT and the mutant.

### Structural modeling of predicted deleterious variants

**Fig. 2:**
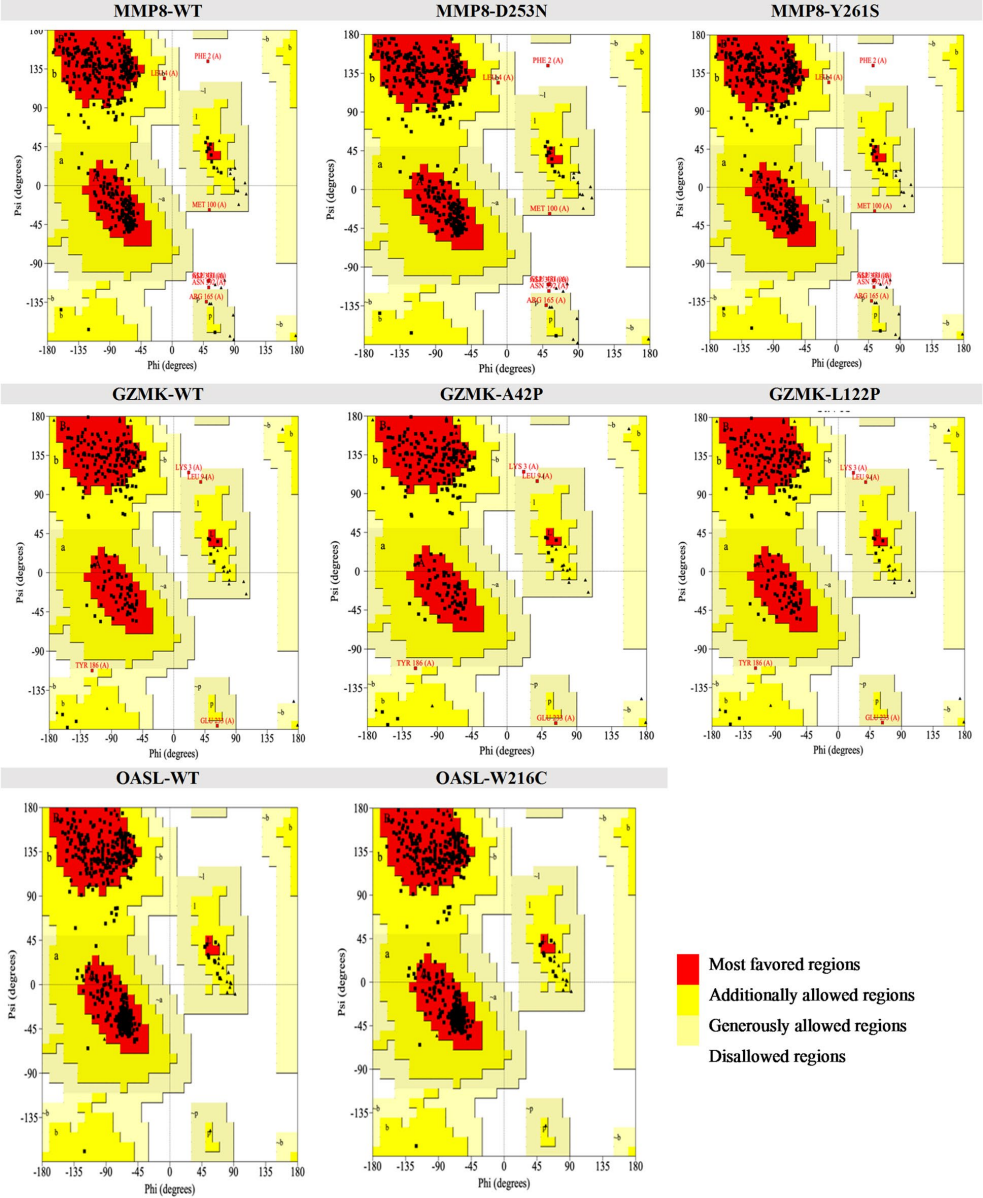
The Ramachandran plots native and mutant models of MMP8, GZMK, and OASL proteins.

AlphaFold was used to generate three-dimensional protein models of MMP8, GZMK, and OASL, and missense mutations were inserted digitally using the PyMoL mutagenesis tool. The structural quality of both WT and mutant models was evaluated using Verify 3D, ERRAT, and PROCHECK. The Verify 3D scores for MMP8 ranged from 88.22% (Y261S) to 89.29% (D253N), indicating continuous agreement between the atomic model and its amino acid sequence. In every case, ERRAT scores remained above 84; Ramachandran plot analysis indicated that 88.1% of residues were located in the most preferred regions, with only 1.0% in forbidden regions across all variants, implying no substantial deviation from anticipated backbone geometry. ERRAT remained persistently elevated at 95.07; however, GZMK exhibited a slight decline in Verify 3D for the L122P mutant (76.52%) compared to the wild type and A42P (78.79%). The PROCHECK study indicated adequate stereochemistry, with fewer than 0.5% of residues located in forbidden regions. OASL exhibited no residues in prohibited regions of the Ramachandran plot; both the WT and W216C mutant adhered to identical validation standards, featuring a Verify 3D score of 85.60% and an ERRAT score of 95.56. While localized conformational alterations cannot be excluded, our findings collectively indicate that the modeled substitutions preserve global protein architecture and do not induce substantial structural disturbances (Table 5, and Fig. 2). These validation metrics collectively confirm that the generated models are of sufficient quality and reliability to serve as robust starting points for subsequent docking and molecular dynamics simulations.

**Table 5:** Structural validation of WT and mutant protein models.

Protein	Model	Verify 3D	ERRAT	PROCHECK			
				Residues in most favored regions	Residues in additional allowed regions	Residues in generously allowed regions	Residues in disallowed regions
MMP8	WT	88.87%	88.7872	88.1%	10.2%	0.7%	1.0%
	D253N	89.29%	84.897	88.1%	10.2%	0.7%	1.0%
	Y261S	88.22%	88.7872	88.1%	10.2%	0.7%	1.0%
GZMK	WT	78.79%	95.0673	82.3%	15.9%	1.3%	0.4%
	A42P	78.79%	95.0673	82.2%	16.0%	1.3%	0.4%
	L122P	76.52%	95.0673	82.2%	16.0%	1.3%	0.4%
OASL	WT	85.60%	95.5556	91.4%	8.6%	0.0%	0.0%
	W216C	85.60%	95.5556	91.4%	8.6%	0.0%	0.0%

### Normal mode analysis of high-risk missense variants

iMODS is a user-friendly interface designed for normal mode analysis, offering extensive data on mobility (B-factors), covariance mapping, protein deformability, and eigenvalues. The eigenvalue signifies cumulative mean square fluctuations and indicates the energy required to deform a structure. Proteins with lower eigenvalues are more easily deformed. This characteristic is crucial for understanding protein dynamics and interactions, as more flexible proteins are generally better suited to adapt to various molecular environments. The iMODS analysis revealed that all selected mutations resulted in a slight decrease in the lowest eigenvalues when compared to their respective WT proteins. This minor reduction indicates a possible alteration in the structural dynamics or flexibility of the selected proteins MMP8, GZMK and OASL (Supplementary Figs. 1, 2, and 3). The DCCMs produced by the iMODS server illustrate significant differences in residue mobility patterns between WT and mutant protein structures. The D253N and Y261S variants in MMP8 alter the intrinsic correlation landscape, with the Y261S mutation showing an increase in positively correlated movements, which suggests enhanced structural coherence or rigidity. When comparing the GZMK protein to the native, the A42P and L122P mutations lead to increased inter-residue correlations, particularly in core domains, indicating potential stability or reduced flexibility. Additionally, the W216C mutation in OASL significantly reduces the long-range anti-correlated motions observed in the native, suggesting a disruption of inherent dynamic interactions. These findings highlight the structural implications of specific point mutations, which can impact protein flexibility and intramolecular interactions and potentially affect functional activity (Supplementary Table 1).

### Molecular docking analysis

**Fig. 3:**
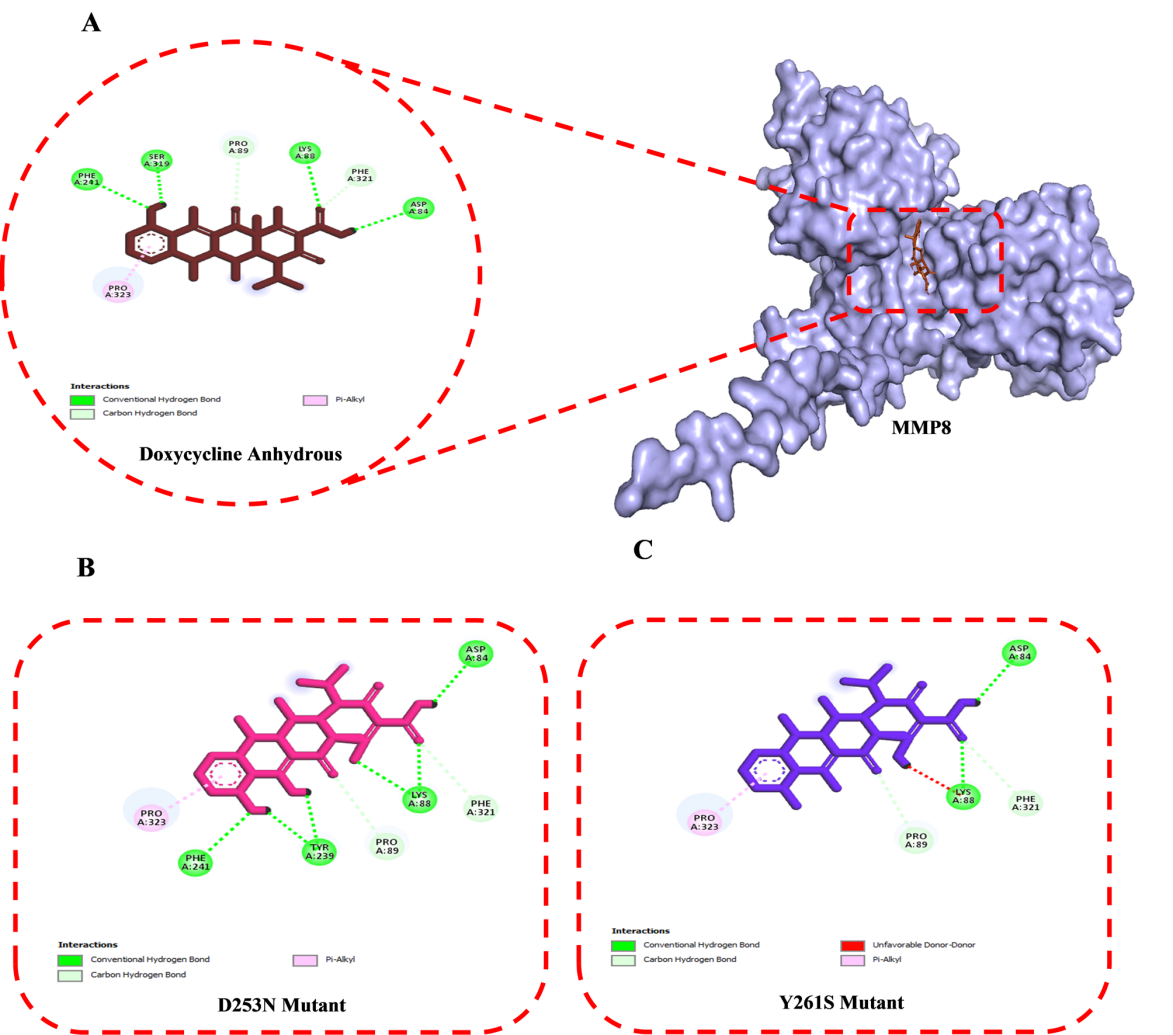
2D intercation diagrams of doxycycline anhydrous with MMP8 proteins. (**A**) WT-MMP8, (**B**) D253N mutant, (**C**) Y261S mutant.

Protein-ligand molecular docking was performed using the CB-Dock2 tool on the 3D structures of the modeled proteins MMP8, GZMK, and OASL (both native and mutant) with the ligands doxycycline anhydrous, bosutinib, and astilbin, respectively. Molecular docking simulations indicated a favorable binding of anhydrous doxycycline to both the native and mutant variants of MMP8. The WT MMP8-doxycycline complex exhibited the highest binding affinity, with a score of − 8.9 kcal/mol. The D253N and Y261S mutants showed slightly lower affinities, both measuring − 8.8 kcal/mol. Key interacting residues in all complexes included Asp84, Lys87, Lys88, Pro89, Asn177, Ser206, Ala207, Asn208, Tyr209, Tyr239, Ala240, Phe241, Ser319, Leu320, Phe321, Trp322, Pro323, Ser324, Leu352, Tyr355, Asp356, and Ile357, suggesting a conserved binding mechanism across all variants (Table 6, Supplementary Table 2). The 3D binding interactions, as shown in the supplementary Table 2, demonstrate that the doxycycline anhydrous maintains stability within a conserved binding pocket across all three complexes, characterized by consistent hydrogen bonding and hydrophobic interactions. The WT-MMP8-doxycycline anhydrous interaction was shown to form six H-bonds with Asp84, Lys88, Pro89, Phe241, Ser319, and Phe321 alongside one other Pi-alkyl interaction with residue Pro323 (Fig. 3A). The D253N-doxycycline anhydrous complex formed six conventional hydrogen bonds with residues Asp84, Lys88, Pro89, Tyr239, Phe241, and Phe321, along with one Pi-alkyl interaction involving Pro323 (Fig. 3B). The Y261S–doxycycline anhydrous complex established four hydrogen bonds with the amino acids Asp84, Lys89, Pro89, and Phe321. Additionally, one Pi-alkyl interaction involved Pro323 (Fig. 3C).

**Fig. 4:**
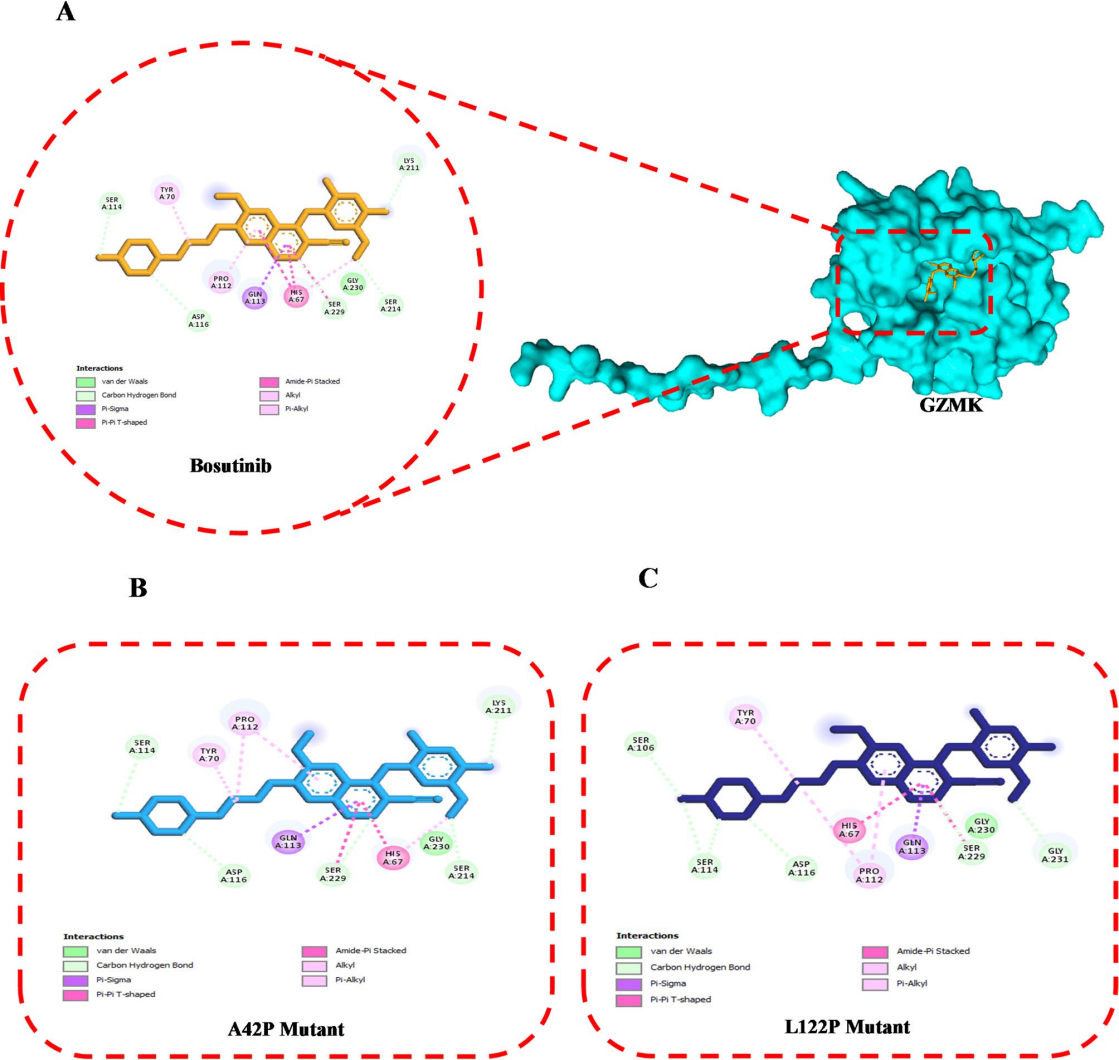
2D intercation diagrams of doxycycline anhydrous with GZMK proteins. (**A**) WT-GZMK, (**B**) A42P mutant, (**C**) L122P mutant.

The docking of bosutinib to GZMK and its variants demonstrated moderate binding affinities. The WT-GZMK complex exhibited the highest affinity at − 7.9 kcal/mol, followed by the A42P mutant at − 7.7 kcal/mol and the L122P mutant at − 7.5 kcal/mol. Binding pockets were primarily located around residues such as Ala66, His67, Tyr70, Arg71, Ser106, Arg107, Val108, Thr109, Asp111, Pro112, Gln113, Ser114, Asn115, Ile117, Asp190, Cys210, Lys211, Gly212, and Ser214. This finding indicates a largely conserved cavity with minor structural changes induced by the mutations (Table 6, Supplementary Table 2). The 3D binding interactions, detailed in Supplementary Table 2, confirm that bosutinib consistently binds within a moderately conserved pocket across both WT and mutant forms of GZMK. The WT-GZMK-bosutinib complex formed five hydrogen bonds with the residues Ser114, Asp116, Lys211, Ser214, and Ser229. Additionally, it exhibited two pi-alkyl interactions with Tyr70 and Pro112, one van der Waals bond with Gly230, one amide-pi stacked interaction with His67, and one pi-sigma bond with Gln113 (Fig. 4A). In the A42P mutant complex, bosutinib formed five hydrogen bonds with the residues Ser114, Asp116, Lys211, Ser214, and Ser229. It also created two pi-alkyl bonds with Tyr70 and Pro112, one amide-pi stacked bond with His67, one pi-sigma interaction with Gln113, and one van der Waals bond with Gly230 (Fig. 4B). The L122P-bosutinib complex established five hydrogen bonds with the following residues: Ser106, Ser114, Asp116, Ser229, and Gly231. Additionally, it formed two pi-alkyl interactions with Tyr70 and Pro112, one amide-pi stacked bond with His67, one pi-sigma bond with Gln113, and an additional van der Waals bond with Gly230 (Fig. 4C).

**Fig. 5:**
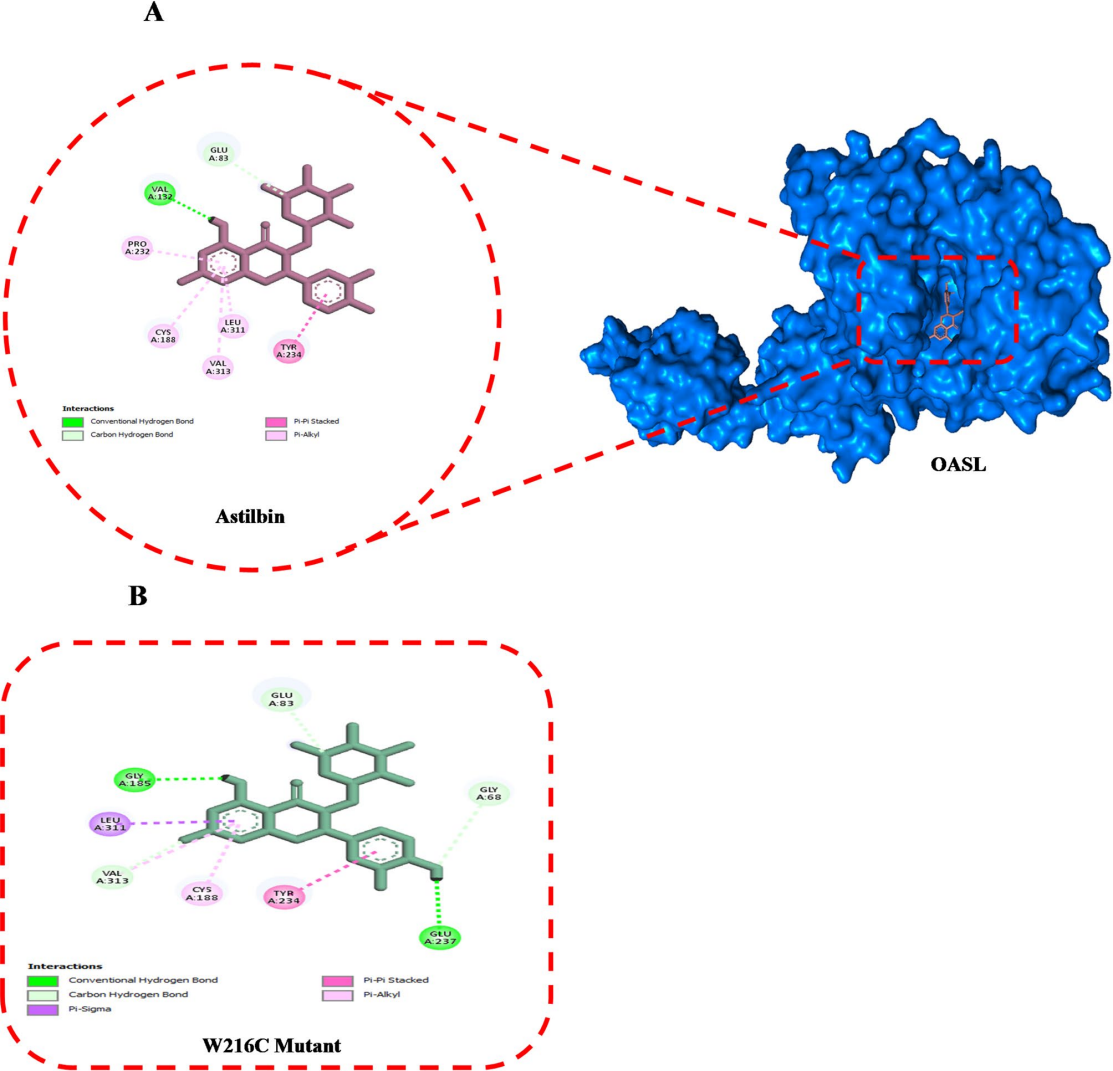
2D intercation diagrams of doxycycline anhydrous with OASL proteins. (**A**) WT-OASL, (**B**) W216C mutant.

Astilbin demonstrated the strongest binding affinity among all tested ligands, particularly with the WT-OASL protein (− 9.3 kcal/mol), while exhibiting a slightly lower binding score for the W216C mutant (− 9.0 kcal/mol). The binding cavity remained consistent across both the native and variant proteins and involved critical interactions with residues such as Val67, Gly68, Ser69, Asn72, Thr74, Glu81, Glu83, Leu84, Val85, Arg131, Val132, Pro133, Ala135, Thr152, Val154, Pro184, Gly185, Asn186, Cys188, Pro189, Phe191, Ser192, Gln195, Arg196, and Val199 (Table 6, Supplementary Table 2). The 3D binding interactions, as detailed in Supplementary Table 2, confirm that astilbin remains stably bound within a conserved binding pocket in both the WT and W216C mutant forms of OASL. In the WT complex, six interactions were identified. These included both conventional and carbon-hydrogen bonds involving key residues such as Glu83 and Val132. Additionally, there were four pi-alkyl interactions with Cys188, Pro232, Leu311, and Val313, as well as one pi-pi stacked bond with Tyr234 (Fig. 5A). In the W216C mutant, astilbin established five interactions, including Pi-alkyl and hydrogen bonds with Gly68, Glu83, Gly185, Cys188, Tyr234, Glu237, and Leu311. This indicates that binding stability is maintained despite the mutation (Fig. 5B).

**Table 6:** Binding affinity and contact residues of WT and mutant proteins (MMP8, GZMK, and OASL) with selected ligands.

Protein	Models	Binding affinity (Kcal/mol)	Contact residues
MMP8	MMP8-WT-Doxycycline Anhydrous	− 8.9	Asp84, Lys87, Lys88, Pro89, Asn177, Ser206, Ala207, Asn208, Tyr209, Tyr239, Ala240, Phe241, Ser319, Leu320, Phe321, Trp322, Pro323, Ser324, Leu352, Tyr355, Asp356, Ile357.
	D253N-Doxycycline Anhydrous	− 8.8	Asp84, Lys87, Lys88, Pro89, Asn177, Ser206, Ala207, Asn208, Tyr209, Tyr239, Ala240, Phe241, Ser319, Leu320, Phe321, Trp322, Pro323, Ser324, Tyr355, Asp356, Ile357.
	Y261S-Doxycycline Anhydrous	− 8.8	Asp84, Lys87, Lys88, Pro89, Asn177, Ser206, Ala207, Asn208, Tyr209, Tyr239, Ala240, Phe241, Ser319, Leu320, Phe321, Trp322, Pro323, Ser324, Leu352, Tyr355, Asp356, Ile357.
GZMK	GZMK-WT-Bosutinib	− 7.9	Ala66, His67, Tyr70, Arg71, Ser106, Arg107, Val108, Thr109, Asp111, Pro112, Gln113, Ser114, Asn115, Asp116, Ile117, Asp190, Cys210, Lys211, Gly212, Ser214, Ser229, Gly230, Gly231, His232, Glu233, Cys234, Lys239, Ile242.
	A42P-Bosutinib	− 7.7	Ala66, His67, Tyr70, Arg71, Ser106, Val108, Thr109, Asp111, Pro112, Gln113, Ser114, Asn115, Asp116, Ile117, Asp190, Cys210, Lys211, Gly212, Ser214, Ser229, Gly230, Gly231, His232, Glu233, Ile242.
	L122P-Bosutinib	− 7.5	Ala66, His67, Tyr70, Arg71, Pro104, Ser106, Val108, Thr109, Asp111, Pro112, Gln113, Ser114, Asn115, Asp116, Ile117, Asp190, Pro191, Phe192, Ser209, Cys210, Lys211, Gly212, Ser214, Val228, Ser229, Gly230, Gly231, His232, Glu233, Cys234, Ile242.
OASL	OASL-WT-Astilbin	− 9.3	Val67, Gly68, Ser69, Asn72, Thr74, Glu81, Glu83, Leu84, Val85, Arg131, Val132, Pro133, Ala135, Thr152, Val154, Pro184, Gly185, Asn186, Cys188, Pro189, Phe191, Ser192, Gln195, Arg196, Val199, Lys214, Lys222, Leu230, Pro231, Pro232, Leu233, Tyr234, Glu237, Asp305, Leu311, Asn312, Val313, Ala314, Tyr317.
	W216C-Astilbin	− 9.0	Val67, Gly68, Ser69, Asn72, Glu81, Glu83, Leu84, Val85, Arg131, Val132, Pro133, Ala135, Thr152, Ile153, Val154, Pro184, Gly185, Asn186, Cys188, Pro189, Ser192, Glu193, Gln195, Arg196, Asn197, Val199, Lys214, Pro231, Pro232, Leu233, Tyr234, Glu237, Leu311, Asn312, Val313, Tyr317.

### Molecular dynamics simulations

#### MMP8 protein and mutants

**Fig. 6:**
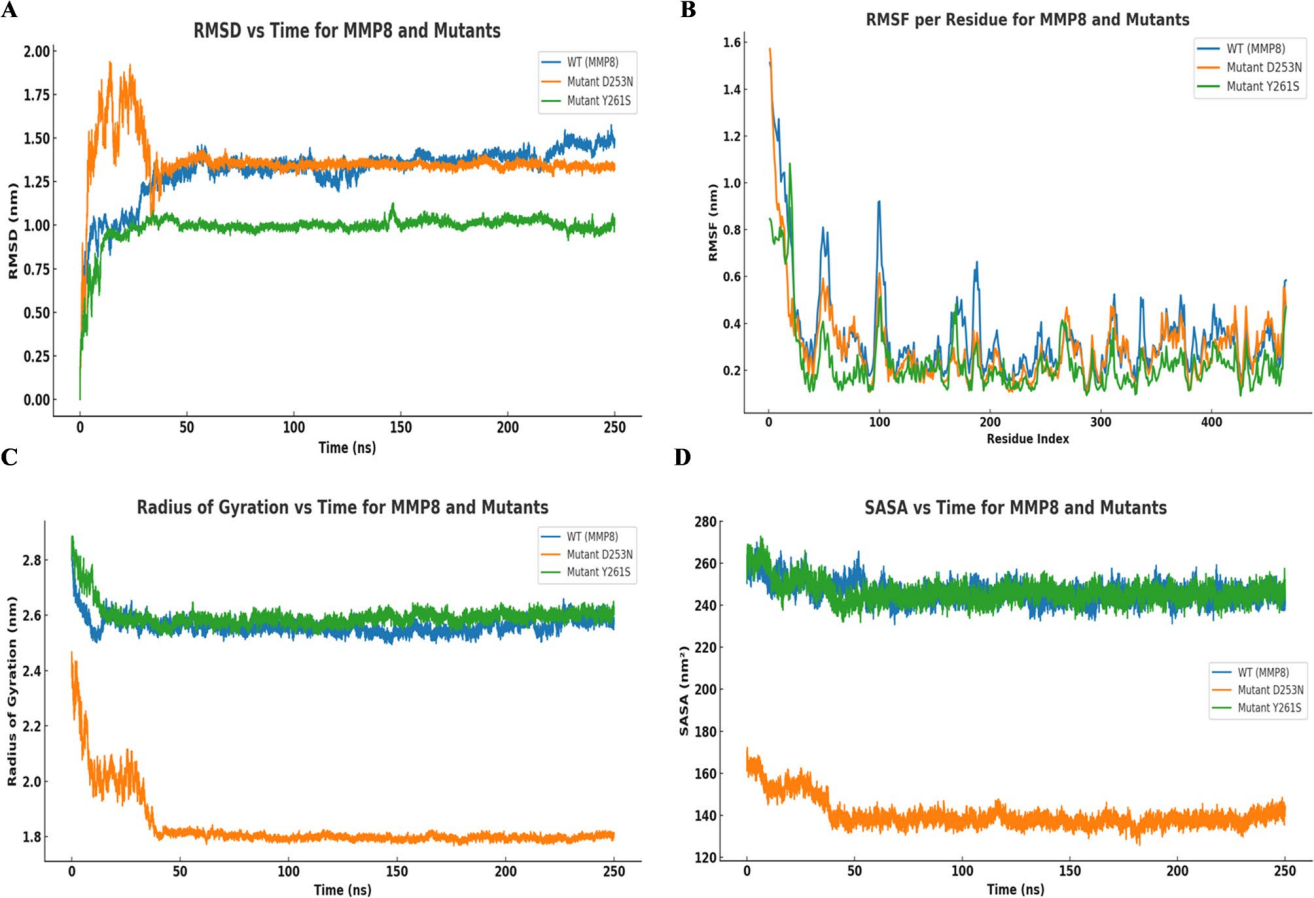
MDS run of 250ns WT-MMP8 and variants (D253N and Y261S) displaying (**A**) RMSD (**B**) RMSF (**C**) Rg and (**D**) SASA.

The RMSD plot compares the structural stability of WT-MMP8 and its mutants D253N and Y261S over a 250 ns simulation. The WT exhibits moderate fluctuations, stabilizing around 1.3–1.4 nm, indicating typical flexibility. The D253N mutant shows an early peak exceeding 1.75 nm, followed by a gradual decline and stabilization near 1.3 nm, suggesting initial instability due to conformational rearrangement. In contrast, the Y261S mutant maintains the lowest RMSD (~ 1.0–1.1 nm), indicating a more stable and less flexible structure. Overall, the Y261S mutation enhances structural stability, while D253N induces transient instability compared to the WT (Fig. 6A).

The RMSF plot shows similar overall fluctuation patterns among WT-MMP8 and the D253N and Y261S variants. All three proteins display high flexibility at the N-terminal region, followed by stabilization across the core of the sequence. The D253N mutant does not exhibit a distinct RMSF peak near the mutation site but shows local fluctuations comparable to the WT. The Y261S variant generally displays slightly reduced fluctuations in several regions, although this reduction is modest and not uniform across the entire sequence, suggesting minor local stabilizing effects rather than a global rigidity increase. (Fig. 6B).

Following that, the system trajectories were analyzed using the Rg to assess their compactness. The WT and Y261S maintain similar profiles throughout the 250 ns trajectory, with Rg values consistently around 2.6 nm, suggesting preservation of an extended and stable tertiary structure. In contrast, the D253N variant undergoes a rapid and sustained decrease in Rg within the first 50 ns, reaching a stable value near 1.8 nm. This pronounced reduction indicates a significant structural compaction, possibly due to altered intramolecular interactions caused by the aspartate-to-asparagine substitution (Fig. 6C).

Afterward, we also computed the solvent-accessible surface area. The SASA plot shows clear distinctions in solvent exposure among WT-MMP8 and its D253N and Y261S variants over 250 ns. The WT and Y261S exhibit comparable SASA values, consistently ranging between 245 and 265 nm², indicating preservation of a relatively open and solvent-exposed structure. In contrast, the D253N mutant undergoes a pronounced decrease in SASA during the early phase of the trajectory, stabilizing near 140 nm². This reduction reflects a substantial decline in molecular surface exposure, likely due to a compaction of the tertiary structure (Fig. 6D).

The results of MD simulation indicate that the D253N mutation in MMP8 leads to measurable structural perturbations, as reflected by increased local atomic fluctuations, reduced global dimensions, and diminished solvent exposure. These deviations suggest a shift toward a more compact and less solvent-accessible conformation, rather than a complete collapse of the native fold. The changes observed across RMSD, RMSF, Rg, and SASA profiles are consistent with a potential reduction in conformational stability, which could ultimately affect the structural integrity and functional properties of MMP8.

#### GZMK protein and mutants

**Fig. 7:**
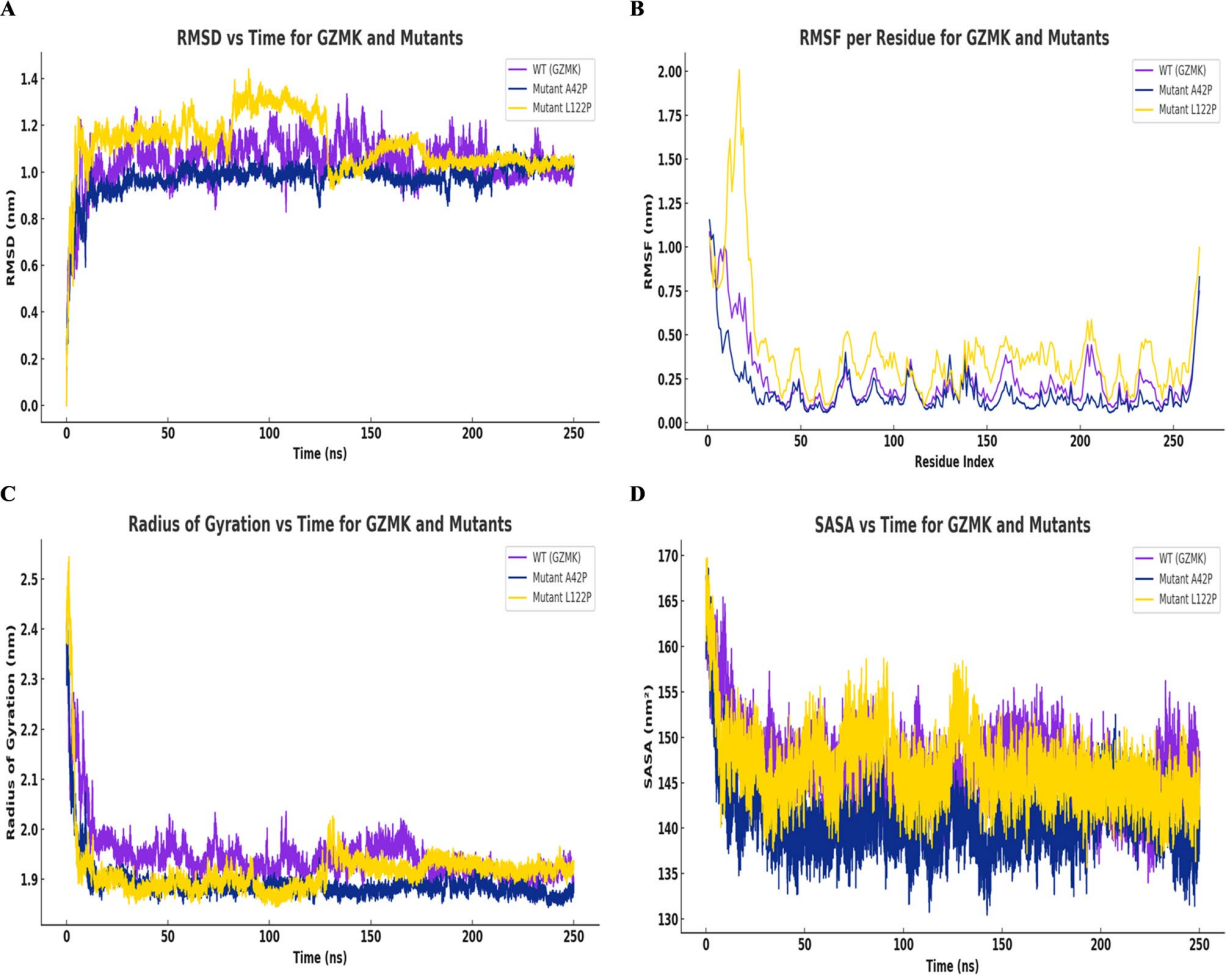
MDS run of 250ns WT-GZMK and variants (A42P and L122P) displaying (**A**) RMSD (**B**) RMSF (**C**) Rg and (**D**) SASA.

Initially, the RMSD profile of GZMK and its variants reveals distinct effects on structural deviation over the course of 250 ns. The WT exhibits a rapid stabilization phase, maintaining an average RMSD near 1.0 nm with limited amplitude, indicative of conserved structural organization. The A42P variant presents a comparable but slightly reduced deviation, suggesting constrained flexibility within the polypeptide chain. In contrast, the L122P substitution results in elevated and more variable RMSD values, particularly during the early and mid-phases of the simulation. This behavior reflects increased structural rearrangements, pointing to a less stable conformation (Fig. 7A).

Subsequently, the RMSF profile of GZMK and its variants highlights residue-specific fluctuations across the polypeptide chain. The WT displays moderate mobility, with elevated values confined to terminal residues, consistent with expected intrinsic disorder in these regions. The A42P variant follows a similar pattern, with minor increases in flexibility at select positions, indicating that the substitution exerts minimal structural disruption. In contrast, the L122P mutant shows a marked increase in atomic fluctuations, particularly at the N-terminal region and several internal segments, with RMSF values exceeding 2.0 nm. This enhanced local mobility suggests altered backbone dynamics and possible destabilization of secondary structural elements (Fig. 7B).

Following that, the Rg analysis highlights differences in molecular dimensions between WT-GZMK and its A42P and L122P variants. The WT maintains Rg values near 2.0 nm, reflecting a relatively expanded conformation with preserved tertiary structure. The A42P substitution leads to a slight decrease in Rg, stabilizing around 1.9 nm, consistent with moderate structural compaction. In contrast, the L122P variant displays persistently lower Rg values, remaining below 1.9 nm throughout the simulation. This observation indicates a denser molecular packing and possible reorganization of structural domains (Fig. 7C).

Thereafter, the SASA analysis of GZMK and its variants reveals differences in solvent exposure associated with each substitution. The WT maintains SASA values between 145 and 155 nm², indicative of a stable balance between buried and exposed residues. The A42P variant shows a persistent reduction in SASA, with values approaching 135 nm², consistent with increased molecular compaction and reduced surface exposure. In contrast, the L122P mutant exhibits higher SASA values, frequently above 150 nm², suggesting a more open conformation and potential disruption of intramolecular packing (Fig. 7D).

These findings suggest that the L122P mutation perturbs the conformational stability and alters the solvent interaction profile of GZMK.

#### OASL protein and W216C mutant

**Fig. 8:**
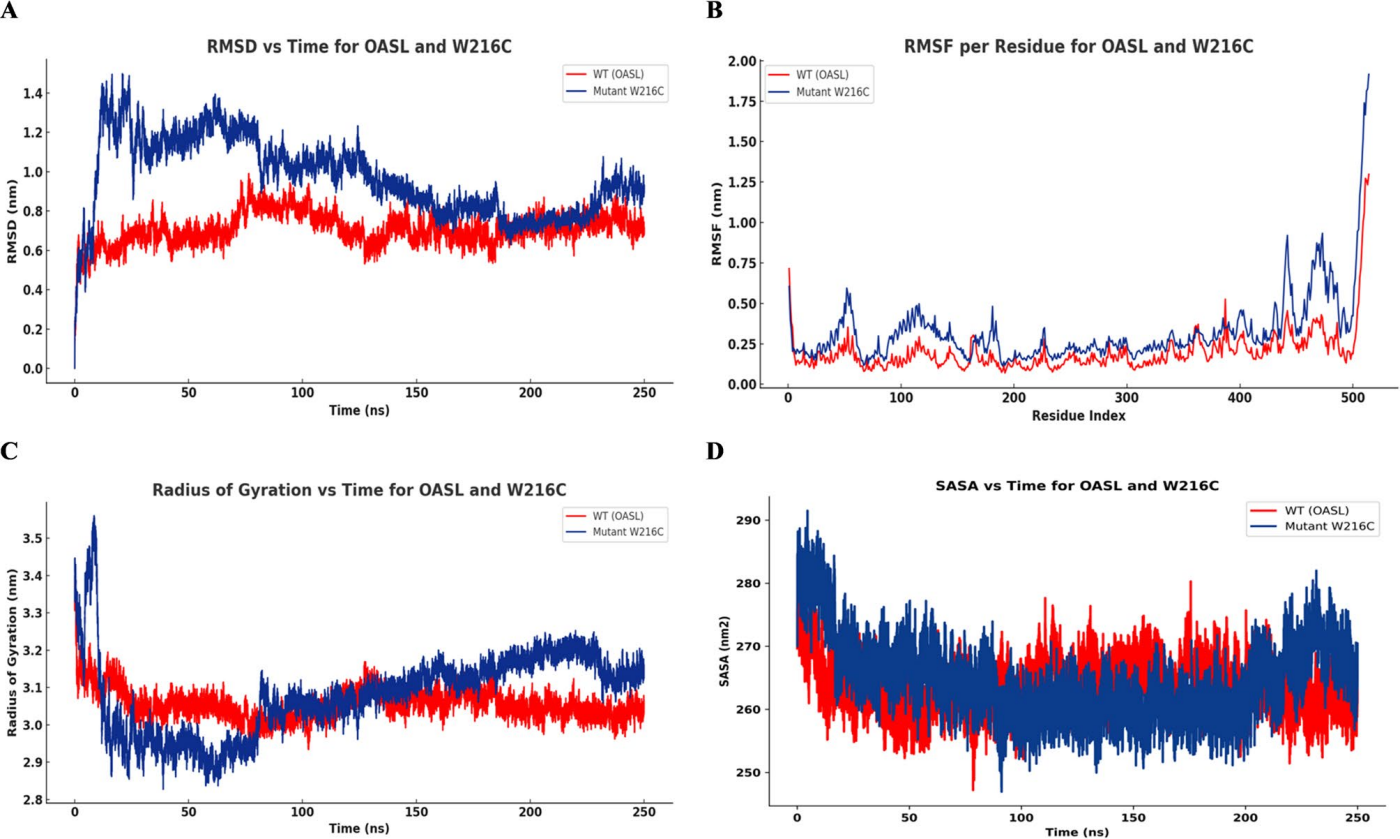
MDS run of 250ns WT-OASL and variant (W216C) displaying (**A**) RMSD (**B**) RMSF (**C**) Rg and (**D**) SASA.

The initial analysis focused on the RMSD analysis of OASL and the W216C variant reveals a significant alteration in structural stability induced by the mutation. The WT protein stabilizes rapidly and maintains RMSD values between 0.7 and 0.8 nm, indicating limited deviation from the initial conformation. In contrast, the W216C mutant displays higher RMSD values throughout the simulation, frequently approaching or exceeding 1.1 nm, particularly during the first 150 ns. This behavior reflects increased conformational variability and reduced structural coherence (Fig. 8A).

Following this, the RMSF analysis highlights distinct differences in residue-level mobility between WT OASL and the W216C variant. In the WT, atomic fluctuations remain low throughout most of the sequence, with values generally under 0.4 nm, reflecting restricted backbone motion. In contrast, the W216C mutant exhibits increased flexibility across several regions, particularly between residues 90–120, 360–400, and at the C-terminal segment beyond residue 500, where fluctuations exceed 1.5 nm. These shifts suggest that the W216C substitution alters local packing or weakens intramolecular restraints, leading to enhanced segmental motion (Fig. 8B).

In the next step, the Rg profile for OASL and the W216C variant demonstrates mutation-induced differences in global structural compactness. The WT maintains consistent values around 3.05 nm throughout the simulation, reflecting a stable and compact tertiary arrangement. In contrast, the W216C mutant exhibits a gradual increase in Rg, reaching approximately 3.15 nm, accompanied by broader fluctuations. This upward shift suggests a relaxation of the overall structure and increased spatial distribution of atomic coordinates (Fig. 8C).

The analysis was then extended to the SASA analysis distinguishes the WT-OASL from the W216C variant in terms of molecular surface exposure. The WT maintains relatively stable values, predominantly within the 260–270 nm² range, reflecting limited variation in solvent interaction. The W216C mutant, however, exhibits consistently elevated SASA values, frequently surpassing those of the WT, and displays broader fluctuation amplitude. This increase in accessible surface area indicates a shift toward a more solvent-exposed conformation, potentially due to displacement of peripheral regions or altered side-chain orientation (Fig. 8D).

Collectively, these results indicate that the W216C mutation disrupts intramolecular packing, promotes structural expansion, and alters surface accessibility.

**Fig. 9:**
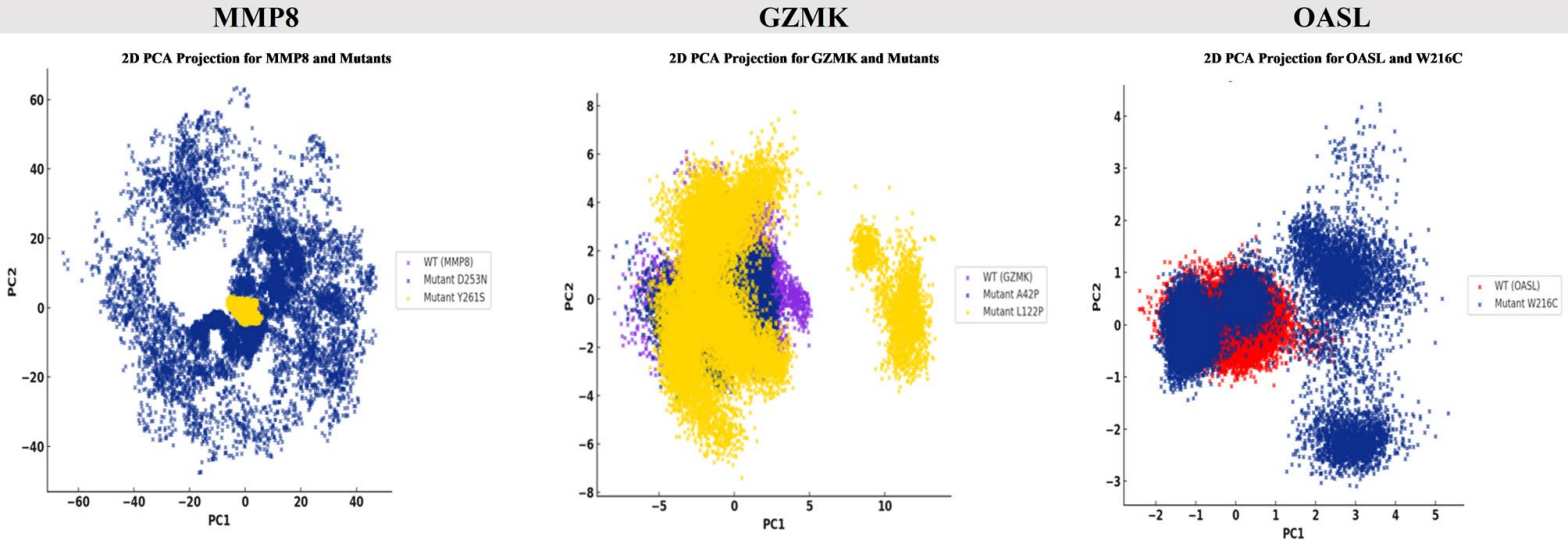
PCA of native MMP8 and mutant structures over 200 ns MDs. (**A**) MMP8: comparison of WT, D253N (blue color) and Y261S (yellow color). (**B**) GZMK: comparison of WT, A42P (blue color) and L122P (yellow color). (**C**) OASL: comparison of WT and W216C (blue color).

To characterize the impact of single-point mutations on conformational dynamics, PCA was applied to the molecular dynamics trajectories of MMP8, GZMK, and OASL. At the initial level, the D253N variant of MMP8 exhibited a broad distribution along the first two principal components, reflecting increased structural heterogeneity compared to the WT ensemble. In contrast, the Y261S variant remained confined to a narrow region, indicating that this substitution preserved the global conformational behavior of the protein (Fig. 9A). In the subsequent comparison, GZMK bearing the L122P mutation showed a clearly displaced and dispersed projection, suggesting an altered dynamic profile and deviation from the native conformational space. Meanwhile, the A42P variant partially overlapped with the WT distribution, implying a more limited structural effect (Fig. 9B). In the final set of projections, the W216C mutation in OASL resulted in the emergence of several distinct conformational clusters, distinct from the compact distribution of the WT, pointing to a mutation-induced reorganization of the structural landscape (Fig. 9C). Furthermore, the PCA results demonstrated that D253N, L122P, and W216C mutants had the greatest increase in conformational space compared to the WT proteins and all other variants.

#### Molecular mechanics poisson-boltzmann surface area (MM-PBSA) binding free energy analysis

MM/PBSA binding free energy calculations were performed to quantify the energetic contributions governing ligand binding in the WT and mutant complexes of MMP8, GZMK, and OASL. The results revealed mutation-dependent differences in total binding free energy (ΔG_total) and its individual components (ΔE_vdW, ΔE_ele, ΔG_polar, and ΔG_nonpolar). For MMP8, the WT complex exhibited a favorable binding free energy (ΔG_total = − 14.38 kcal/mol), which was maintained in the Y261S variant (ΔG_total = − 14.09 kcal/mol), whereas the D253N mutation showed a pronounced reduction in binding affinity (ΔG_total = − 4.51 kcal/mol). In the GZMK system, all complexes displayed favorable binding free energies, with values of − 16.94 kcal/mol for the wild type, − 16.37 kcal/mol for A42P, and − 13.32 kcal/mol for L122P, indicating moderate energetic variation among variants. Notably, for OASL, the W216C mutant demonstrated a more favorable binding free energy (ΔG_total = − 19.63 kcal/mol) compared to the wild type (ΔG_total = − 13.40 kcal/mol). These results are summarized in Supplementary Tables 3 and illustrated in Supplementary Fig. 4.

## Discussion

Single nucleotide polymorphisms (SNPs) represent an important source of genetic variability and can significantly influence protein structure, stability, and function, thereby contributing to interindividual differences in disease susceptibility and clinical outcome^53,54^. Missense variants are particularly relevant, as they may induce local or global conformational changes that affect thermodynamic stability, molecular interactions, and functional performance. Given the practical limitations of large-scale experimental characterization, computational approaches have become indispensable for prioritizing potentially deleterious variants and systematically exploring genotype–phenotype relationships^55,56^.

Recent studies have further highlighted the contribution of integrative computational methods to the understanding of host–virus interactions and immune-related molecular mechanisms. In silico investigations of SARS-CoV-2 spike protein variants have demonstrated how specific mutations can alter structural stability, conformational dynamics, and interaction behavior, thereby modulating viral infectivity and host responses^57^. Similarly, structure-based computational analyses of immune mediators such as interleukin-18 have underscored the utility of molecular modeling and molecular dynamics (MD) simulations for characterizing protein flexibility, stability, and interaction networks relevant to antiviral defense and immune signaling pathways^58^. These findings support the application of comprehensive in silico frameworks, such as the one adopted in the present study, to investigate the molecular consequences of genetic variation in host proteins implicated in epidemic viral infections.

In this work, we applied a multi-level computational strategy to investigate selected missense variants in MMP8, GZMK, and OASL, three proteins involved in immune regulation and host responses to viral infections. By integrating sequence-based pathogenicity prediction, protein stability assessment, structural dynamics analysis, molecular docking, MD simulations, and MM/PBSA binding free energy calculations, we provide a detailed evaluation of mutation-induced structural and energetic perturbations beyond static modeling approaches.

For MMP8, the D253N and Y261S variants were consistently predicted as deleterious and destabilizing and were located within evolutionarily conserved regions, indicating their potential functional importance. MD simulations revealed striking differences in the dynamic behavior of these variants. The D253N substitution induced pronounced structural perturbations, as reflected by increased atomic fluctuations, reduced radius of gyration, and decreased solvent-accessible surface area, suggesting a shift toward a more compact yet destabilized conformation. Principal component analysis further demonstrated that D253N broadened the conformational space explored during the simulation, indicative of an altered dynamic equilibrium. In contrast, the Y261S variant maintained restricted motion and global dimensions comparable to the wild-type protein.

These observations were supported by MM/PBSA binding free energy calculations, which revealed a substantial reduction in binding affinity for the D253N variant (ΔG_total = − 4.51 kcal/mol) compared with the wild-type complex (ΔG_total = − 14.38 kcal/mol), primarily due to the loss of favorable electrostatic contributions. In contrast, the Y261S variant retained binding energetics comparable to the wild type (ΔG_total = − 14.09 kcal/mol). These findings suggest that residue D253 is critical for maintaining energetically favorable intermolecular interactions required for stable binding in MMP8.

The functional relevance of MMP8 mutations has been documented in several pathological contexts. Somatic alterations in MMP8 have been linked to impaired enzymatic activity and loss of tumor-suppressive functions in melanoma^59^, while additional studies have highlighted protective roles of MMP8 in tissue homeostasis and inflammation^60^. Consistent with these observations, the pronounced destabilization and reduced binding affinity associated with the D253N variant observed here may compromise protease-mediated signaling pathways involved in immune regulation and host defense during viral infections.

For GZMK, both A42P and L122P variants were predicted to be deleterious and destabilizing and were located at conserved residues. MD analyses indicated that the L122P substitution induced more pronounced conformational perturbations than A42P, including increased backbone fluctuations, reduced compactness, and elevated solvent exposure. Despite these structural changes, MM/PBSA calculations showed only moderate differences in binding free energy among the GZMK complexes (WT: −16.94 kcal/mol; A42P: −16.37 kcal/mol; L122P: −13.32 kcal/mol), suggesting partial preservation of ligand binding. This apparent energetic robustness may reflect compensatory interactions within the binding interface or intrinsic flexibility of the ligand-recognition mechanism. Given emerging evidence linking GZMK-expressing CD8⁺ T cells to chronic inflammation and immune-mediated tissue damage^61^, such mutation-induced alterations in protein dynamics could still influence downstream effector functions.

In the case of OASL, the W216C variant was consistently predicted as deleterious and localized within a highly conserved region. MD simulations revealed substantial deviations in global dynamics, including increased RMSD, broadened RMSF profiles, and expansion of the molecular envelope, indicative of reduced structural cohesion. These findings are in line with previous functional studies showing that rare OASL variants can disrupt interferon-mediated antiviral responses and innate immune signaling^62^. Interestingly, MM/PBSA analysis indicated a more favorable binding free energy for the W216C mutant (ΔG_total = − 19.63 kcal/mol) relative to the wild-type complex (ΔG_total = − 13.40 kcal/mol), driven by enhanced van der Waals and electrostatic interactions. This decoupling between global stability and binding affinity suggests that structural reorganization or increased flexibility may facilitate more favorable intermolecular contacts. However, such destabilization could impair critical protein–protein interactions required for effective antiviral signaling, consistent with reports linking altered interferon pathways to immune dysregulation^63^.

Collectively, these findings demonstrate that protein stability, conformational dynamics, and binding affinity are not necessarily directly correlated. Destabilizing mutations may weaken binding, preserve ligand recognition, or even enhance binding energetics, depending on the balance between structural integrity, flexibility, and energetic contributions. The integration of MD simulations with MM/PBSA analyses therefore provides robust energetic validation of docking-based predictions and captures mutation-dependent effects not accessible through docking alone.

Although the present study offers mechanistic insights into the potential functional impact of nsSNPs in MMP8, GZMK, and OASL, it remains computational in nature. Experimental validation using biochemical and cellular assays will be essential to confirm these predictions and to elucidate the precise contribution of these variants to immune dysregulation and disease susceptibility during epidemic viral infections.

## Conclusion

In summary, this study explored the structural and functional consequences of high-risk missense variants in the MMP8, GZMK, and OASL genes, all of which have relevance in immune response pathways implicated in epidemic viral infections. Through an integrative computational framework, the impact of amino acid substitutions on protein conformation, stability, and potential biological function was systematically evaluated. These findings provide a foundation for targeted experimental validation and may inform future investigations into host-pathogen interactions and genetic susceptibility to viral disease. To guide experimental efforts, we prioritize the following high-risk variants for testing, based on convergent computational evidence: *MMP8* (D253N, Y261S), *GZMK* (A42P, L122P), and *OASL* (W216C).

## Supplementary Information

Below is the link to the electronic supplementary material.

Supplementary Material 1

## Data Availability

The datasets generated and/or analyzed for MMP8, GZMK, and OASL during the current study are available in the Ensembl Genome Browser at the following links: MMP8 ( [https://www.ensembl.org/Homo_sapiens/Gene/Variation_Gene/Table? db=core; g=ENSG00000118113;*r*=11:102711796-102727050](https:/www.ensembl.org/Homo_sapiens/Gene/Variation_Gene/Table? db=core; g=ENSG00000118113;*r*=11:102711796-102727050) ), GZMK ( [https://www.ensembl.org/Homo_sapiens/Gene/Variation_Gene/Table? db=core; g=ENSG00000113088;*r*=5:55024256-55034570](https:/www.ensembl.org/Homo_sapiens/Gene/Variation_Gene/Table? db=core; g=ENSG00000113088;*r*=5:55024256-55034570) ), and OASL ( [https://www.ensembl.org/Homo_sapiens/Gene/Variation_Gene/Table? db=core; g=ENSG00000135114;*r*=12:121017763-121039246](https:/www.ensembl.org/Homo_sapiens/Gene/Variation_Gene/Table? db=core; g=ENSG00000135114;*r*=12:121017763-121039246) ).

## Abbreviations

MMP8: Matrix metalloproteinase-8
GZMK: Granzyme K
OASL: 2′,5′-Oligoadenylate synthetase-like
HCMV: Human cytomegalovirus
CTL: Cytotoxic T lymphocytes
NK: Natural killer
IBDV: Infectious bursal disease virus
IAV: Influenza A virus
MD: Molecular dynamics
PPI: Protein–protein interactions
SNP: Single nucleotide polymorphisms
nsSNPs: Non-synonymous single nucleotide polymorphisms
WT: Wild-type
NMA: Normal mode analysis
ENCoM: Elastic network contact model
iMODS: iMod server
PME: Particle Mesh Ewald
RMSD: Root mean square deviation
RMSF: Root mean square fluctuation
Rg: Radius of gyration
SASA: Solvent accessible surface area
PCA: Principal component analysis
ΔE_vdW: van der Waals energy
ΔE_ele: Electrostatic energy
ΔG_polar: Polar solvation energy
ΔG_nonpolar: Nonpolar solvation energy
ΔG_total: Total binding free energy
DCCMs: Dynamic cross-correlation matrices
