# Supplementary material for: In silico structural and functional characterization of high-risk missense variants in MMP8, GZMK, and OASL genes associated with epidemic viral infections
Source: Sci Rep. 2026 Mar 10;16:12973. doi: 10.1038/s41598-026-40467-w (PMC13096161; doi:10.1038/s41598-026-40467-w)
Supplement: Supplementary file 1 — Supplementary Material 1 [file 41598_2026_40467_MOESM1_ESM.docx]

**In Silico Structural and Functional Characterization of High-Risk Missense Variants in *MMP8*, *GZMK*, and *OASL* Genes Associated with Epidemic Viral Infections**

**Mohamed Et-tanjaouy ^1^*£, Asmae Saih ^2^*£, Omar Machich ^3^, Abdelkrim Guendouzi^4^, Younes Zaid^5, 6^, Hanaa Abdelmoumen^1^**

*^1^Laboratory of Microbiology and Molecular Biology, Faculty of Sciences of Rabat, Mohammed V University in Rabat, Morocco.*

*^2^Laboratory of Biology and Health, URAC 34, Faculty of Sciences Ben M’Scik Hassan II University of Casablanca, Morocco.*

*^3^Laboratory of Physiology and Physiopathology, Department of Biology, Faculty of Science, Mohammed V University in Rabat, Morocco.*

*^4^Laboratory of Chemistry: Synthesis, Properties and Applications. (LCSPA), Faculty of Sciences, University of Saida - Dr Moulay Tahar, Saida, Algeria.*

*^5^Materials, Nanotechnologies and Environment Laboratory, Department of Biology, Faculty of Sciences, Mohammed V University in Rabat, Rabat, Morocco.*

*^6^Immunology and Biodiversity Laboratory, Department of Biology, Ain Chock Faculty of Sciences, Hassan II University, Casablanca, Morocco.*

£These authors contributed equally to this work

(*) Corresponding authors:

SAIH Asmae: Asmae.saihbio@gmail.com, [Asmae.saih-etu@etu.univh2c.ma](mailto:Asmae.saih-etu@etu.univh2c.ma), asmae.saihbio@gmail.com

Mohamed Et-tanjaouy: mohamed_ettanjaouy@um5.ac.ma; Email address: mettanjaoui@gmail.com

Author’s e-mail addresses:

Mohamed Et-tanjaouy: Institutional email address: mohamed_ettanjaouy@um5.ac.ma; Email address: mettanjaoui@gmail.com;

ORCID ID: <https://orcid.org/0009-0005-2559-7223>

Asmae Saih: Email address: asmae.saihbio@gmail.com; ORCID ID: <https://orcid.org/0000-0002-5487-5684>

Omar Machich: Email address: omar.machich@um5r.ac.ma; ORCID ID: <https://orcid.org/0000-0002-2349-3296>

## Abdelkrim Guendouzi: Email address: [guendouzzi@yahoo.fr](mailto:guendouzzi@yahoo.fr); ORCID ID: <https://orcid.org/0000-0001-9476-0489>

Younes Zaid: Institutional email address: y.zaid@um5r.ac.ma; ORCID ID: <https://orcid.org/0000-0001-8750-9106>

Hanaa Abdelmoumen: Email address: hanabdel10@gmail.com; ORCID ID: <https://orcid.org/0000-0002-1228-4931>

**Supplementary Table 1.** DCCM analysis of residue motions in WT and mutant proteins (MMP8, GZMK, and OASL)

| **MMP8-WT** | **D253N Mutant** | **Y261S Mutant** |
| --- | --- | --- |
| **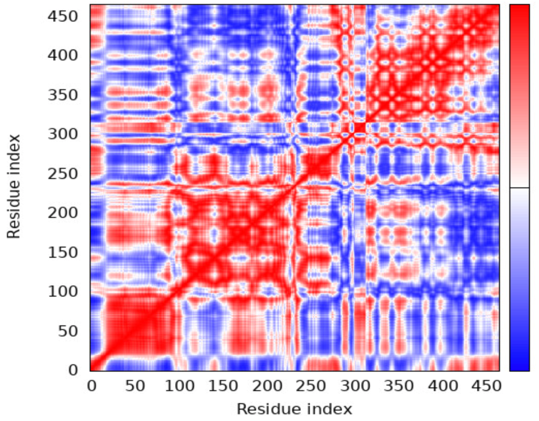** | **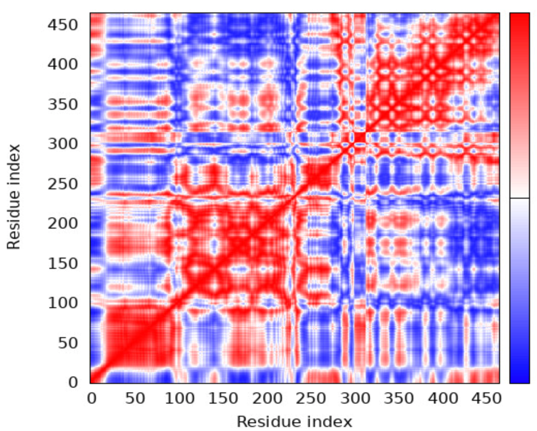** | **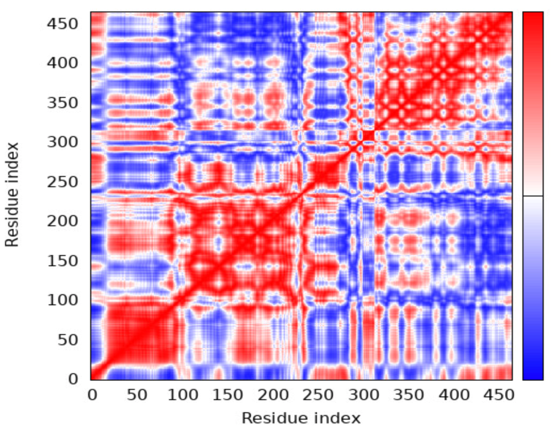** |
| **GZMK-WT** | **A42P Mutant** | **L122P Mutant** |
| **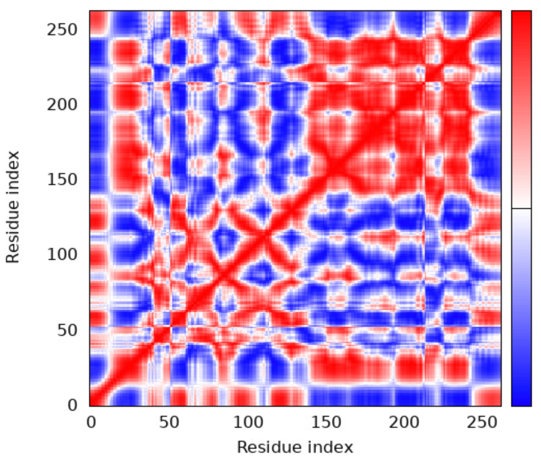** | **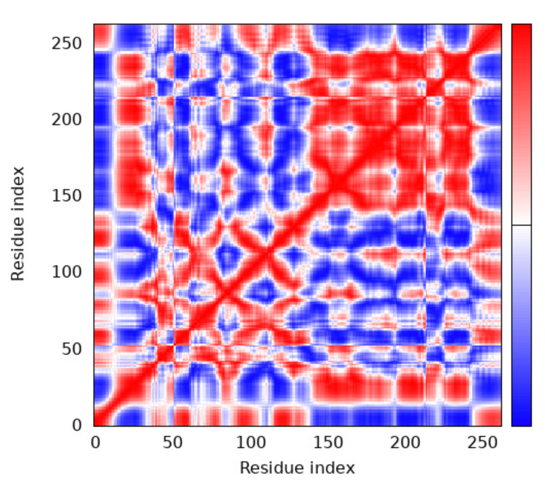** | **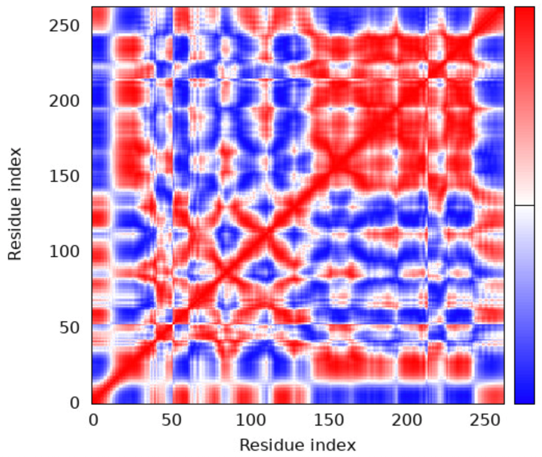** |
| **OASL-WT** | **W216C** |  |
| **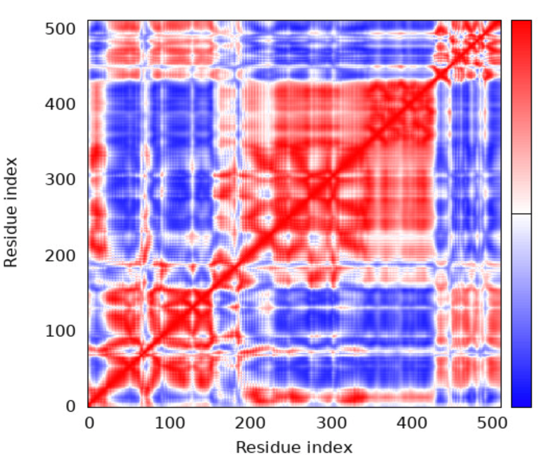** | **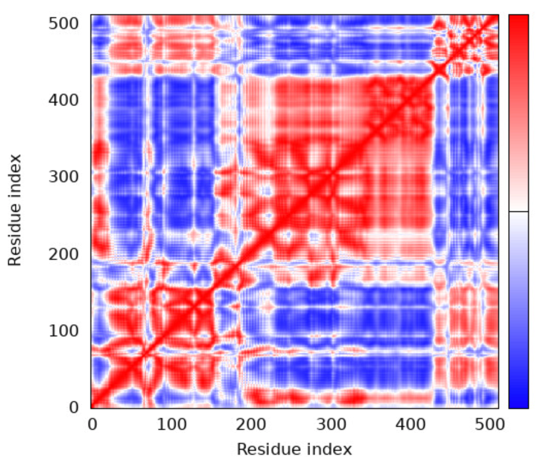** |  |

| **MMP8 Wild-type**  **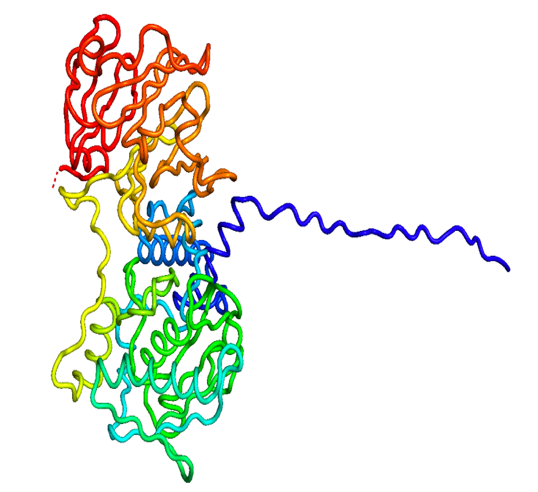**  **A** | **Mutant (D253N)**  **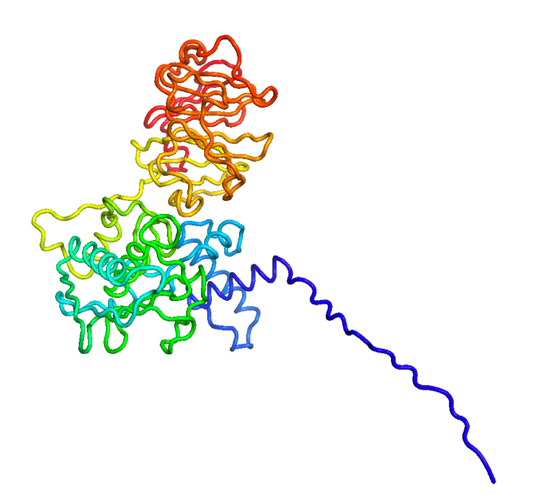** | **Mutant (Y261S)**  **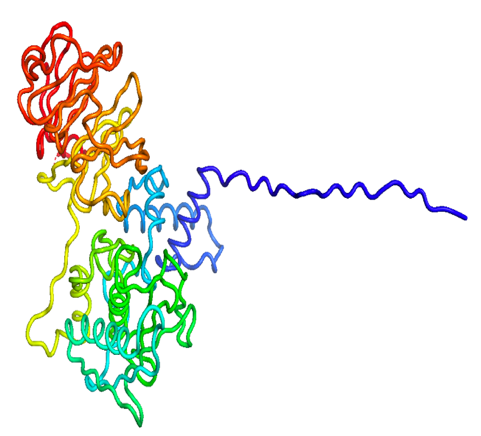** |
| --- | --- | --- |
| 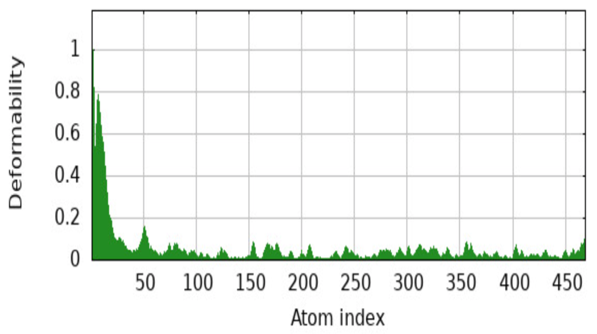  **B** | 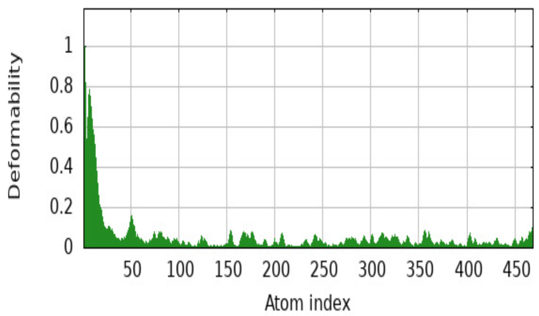 | 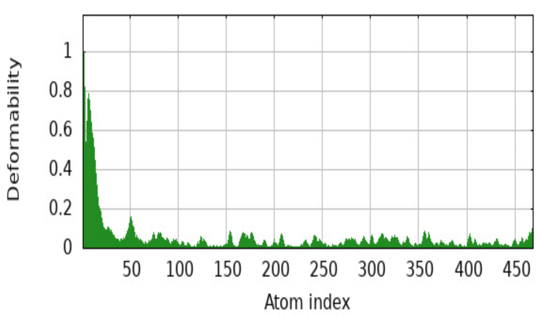 |
| **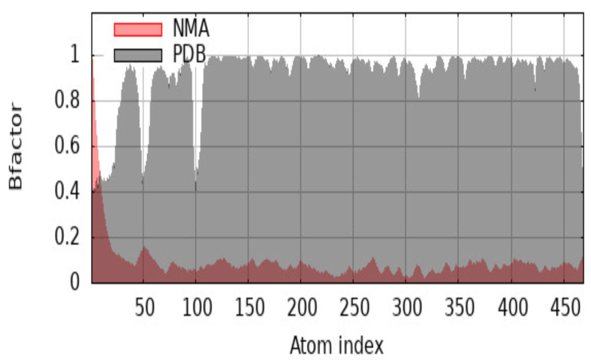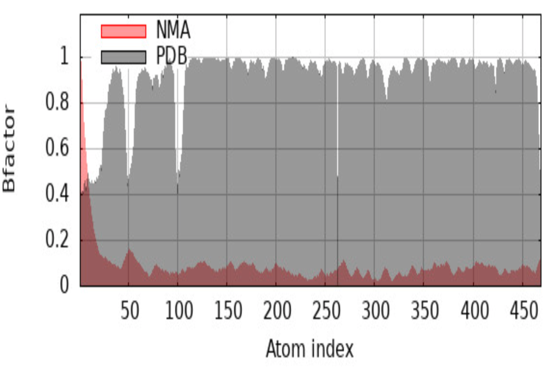 C**  **D** | 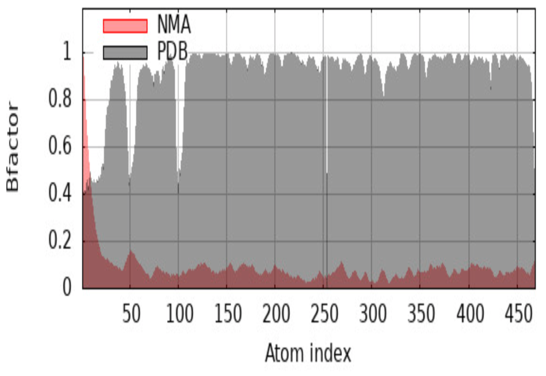 |  |
| 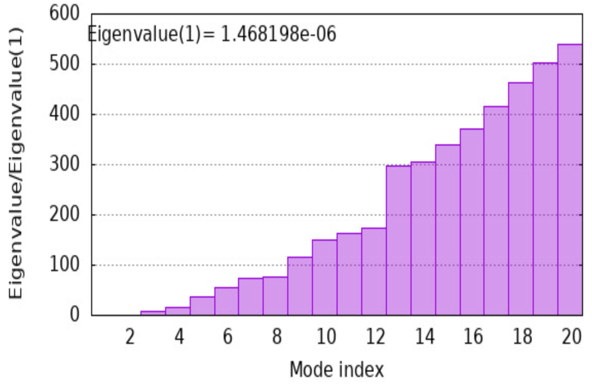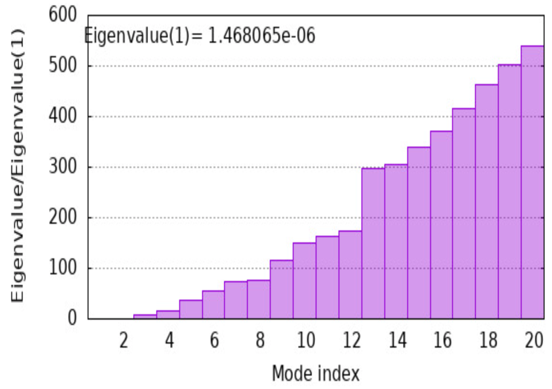 |  | 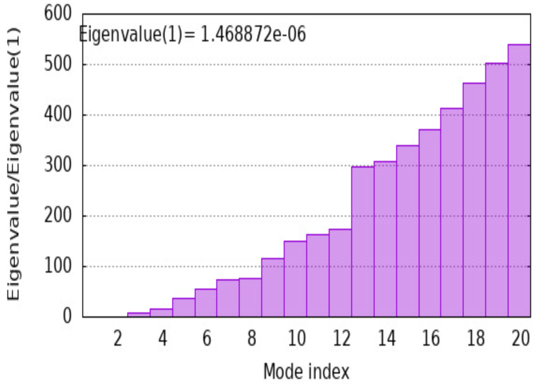 |

**Supplementary Figure 1.** Normal mode analysis of MMP8 WT and mutants (D253N and Y261S) using iMODS server. A. 3D representations of the protein structures (The elongated unstructured tail observed in the wild-type and mutant MMP8 models corresponds to a terminal flexible region lacking stable secondary structure, which is predicted as an extended conformation during modeling and normal mode analysis). B. Deformability plots indicating the flexibility of each residue. C. B-factor comparison plots between theoretical (NMA-derived) and empirical (PDB) fluctuations. D. Eigenvalues plots.

| **GZMK-WT**  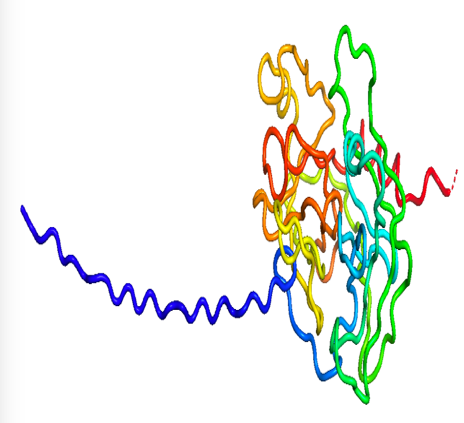 **A** | **A42P Mutant**  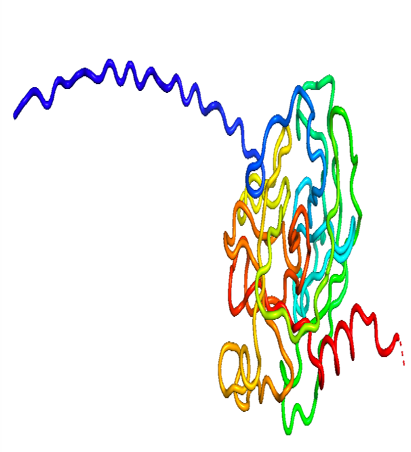 | 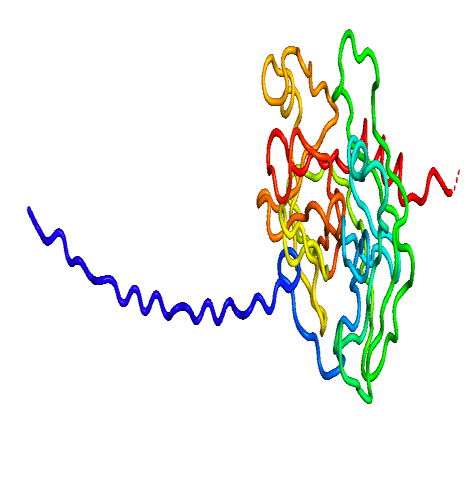**L122P Mutant** |
| --- | --- | --- |
| **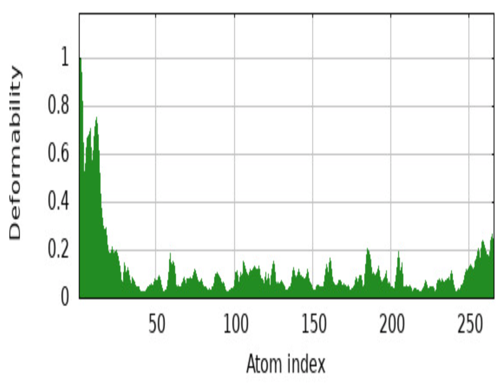 B**  **C** | 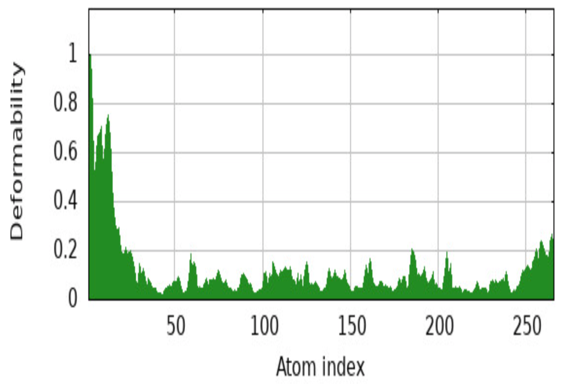 |  |
| 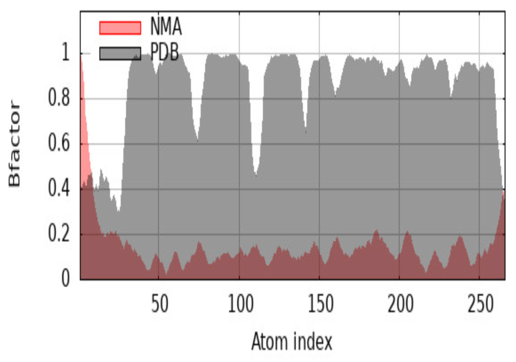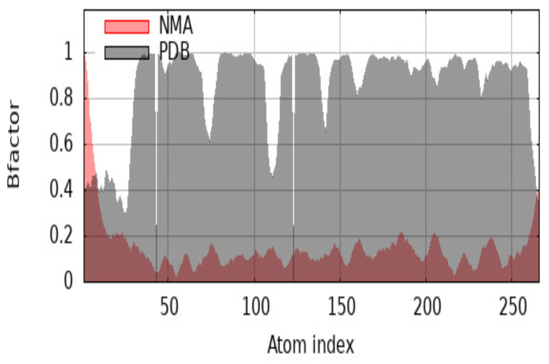  **D** | 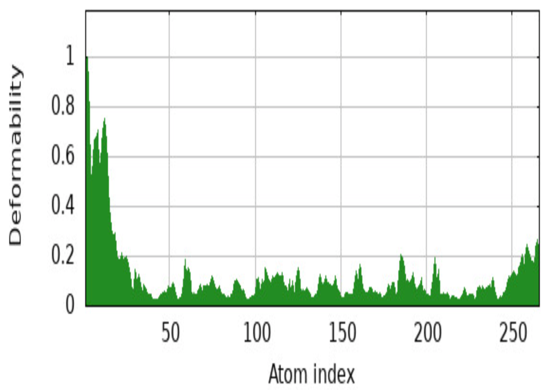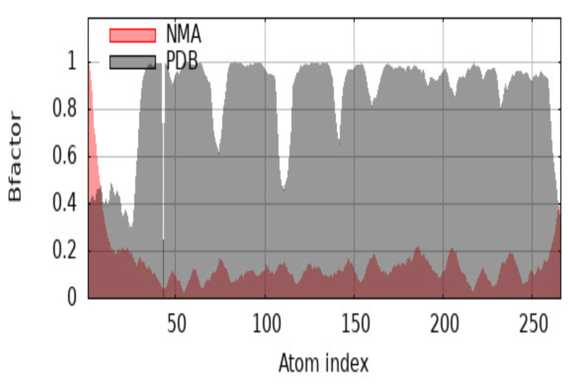 |  |
| 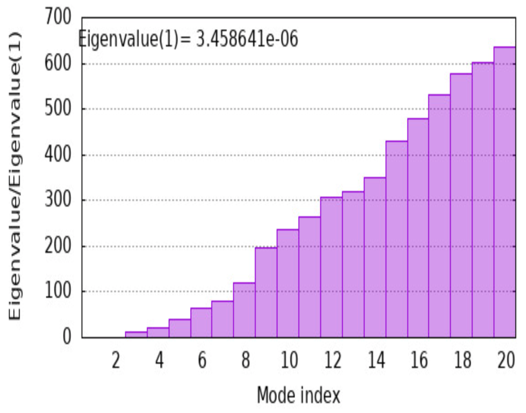 | 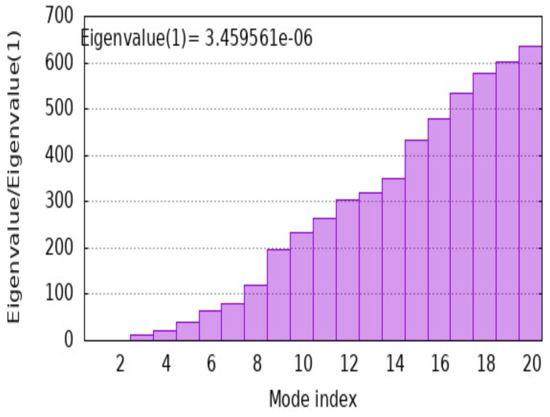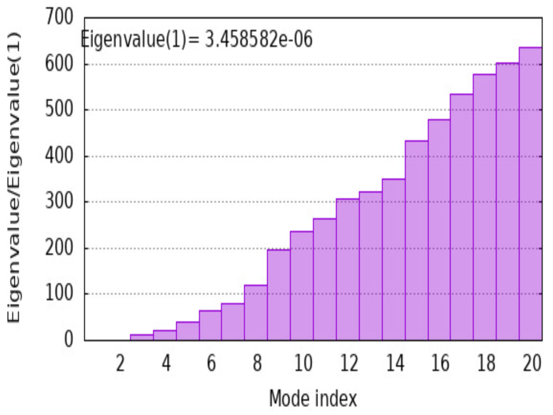 |  |

**Supplementary Figure 2.** Normal mode analysis of GZMK WT and mutants (A42P and L122P) using iMODS server. A. 3D representations of the protein structures. B. Deformability plots indicating the flexibility of each residue. C. B-factor comparison plots between theoretical (NMA-derived) and empirical (PDB) fluctuations. D. Eigenvalues plots.

| **OASL-WT**  **A**  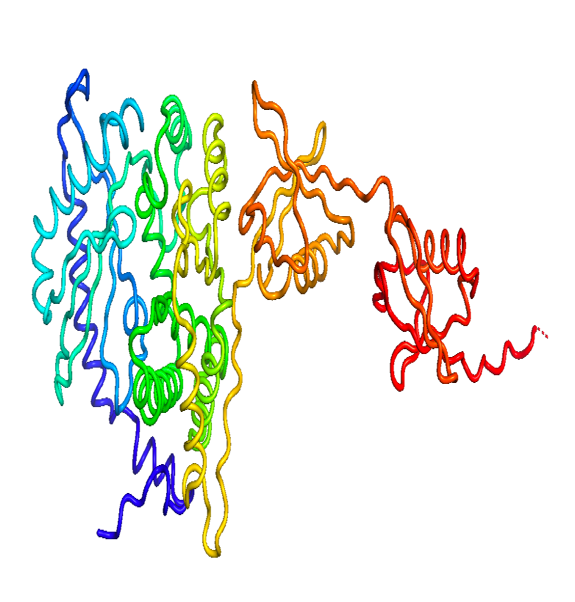 | **W216C Mutant**  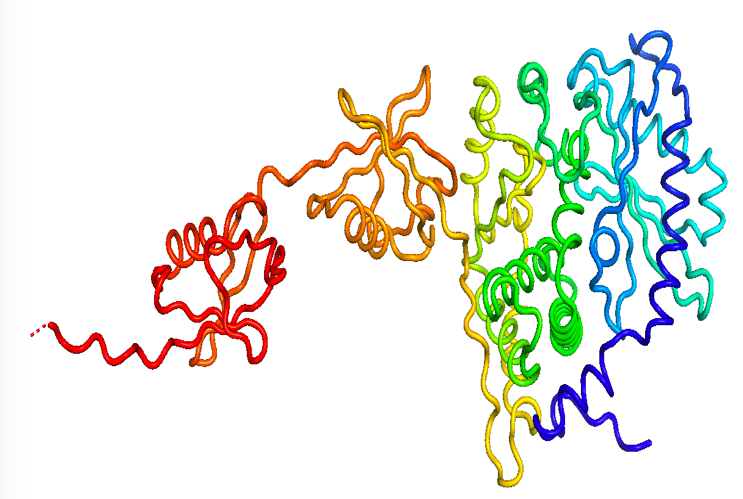 |
| --- | --- |
| **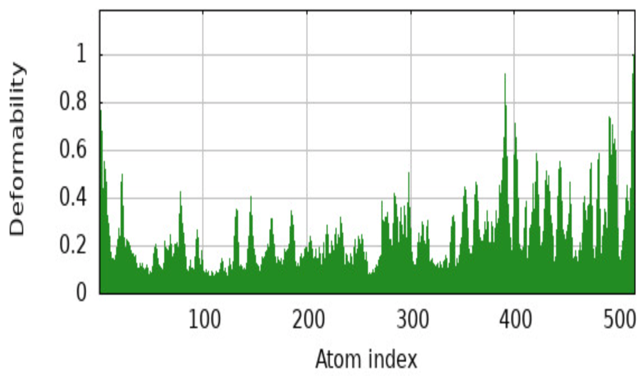 B** | 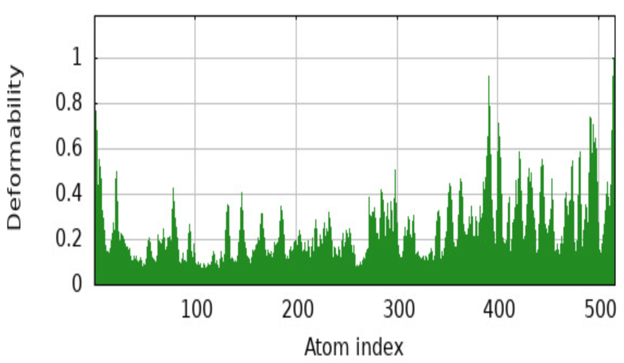 |
| 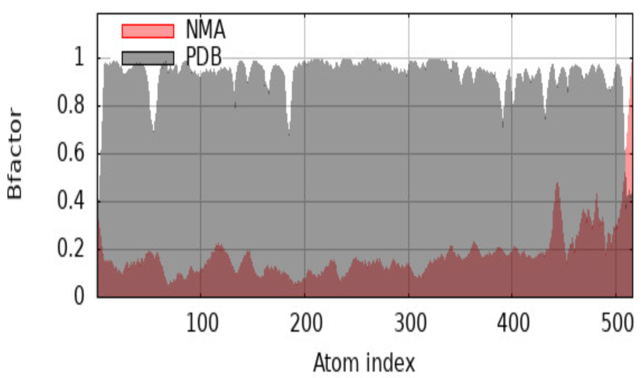  **C** | 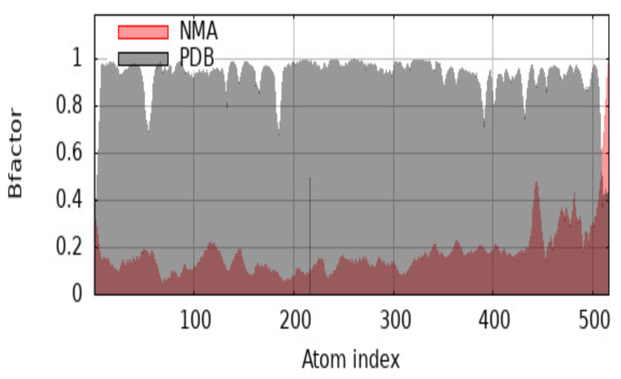 |
| **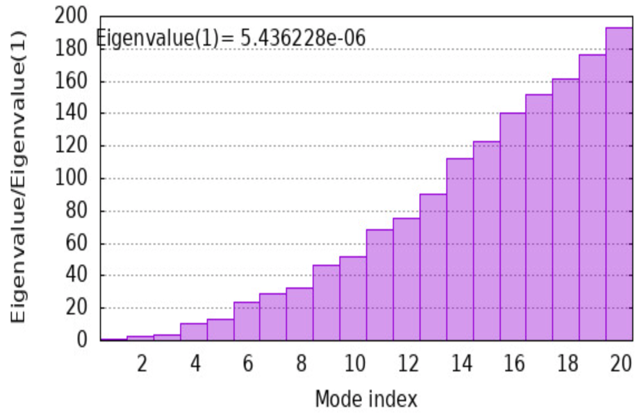 D** | 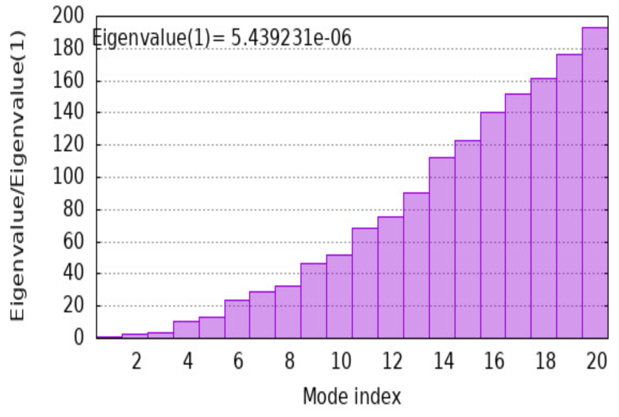 |

**Supplementary Figure 3.** Normal mode analysis of OASL WT and mutants (W216C) using iMODS server. A. 3D representations of the protein structures. B. Deformability plots indicating the flexibility of each residue. C. B-factor comparison plots between theoretical (NMA-derived) and empirical (PDB) fluctuations. D. Eigenvalues plots.

**Supplementary Table 2.** 3D docking poses of native and mutated protein variants with doxycycline, bosutinib, and Astilbin.

| Protein | Models | 3D binding interactions |
| --- | --- | --- |
| MMP8 | MMP8-WT-Doxycycline  Anhydrous | **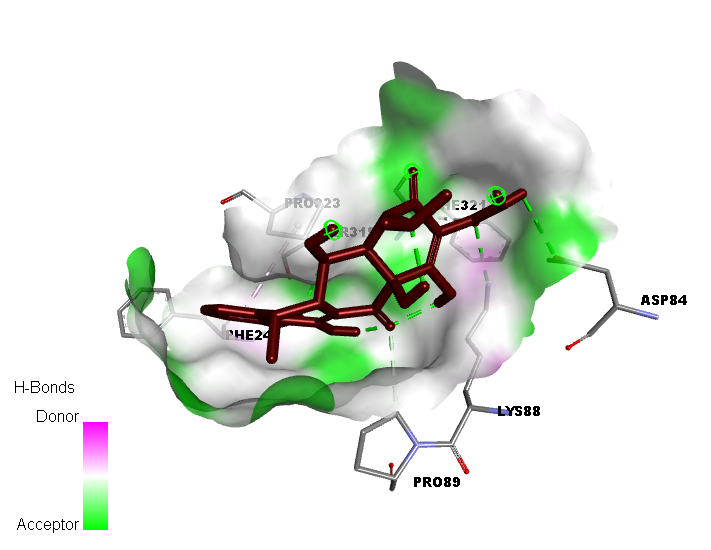** |
|  | D253N-Doxycycline Anhydrous | **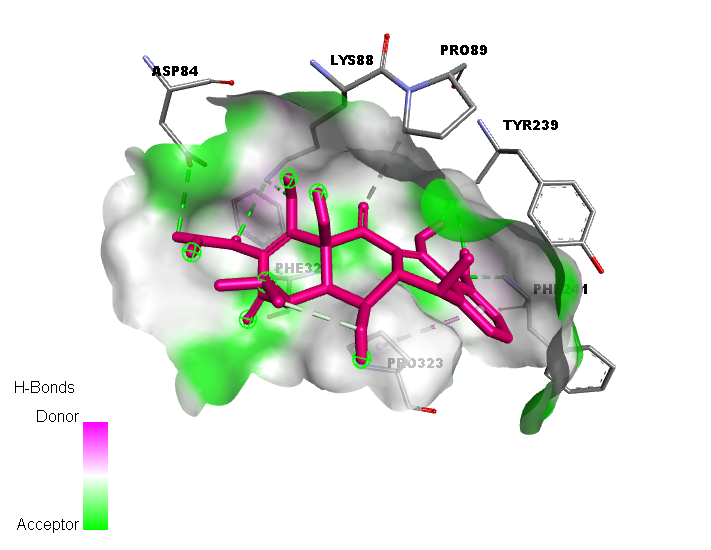** |
|  | Y261S-Doxycycline Anhydrous | **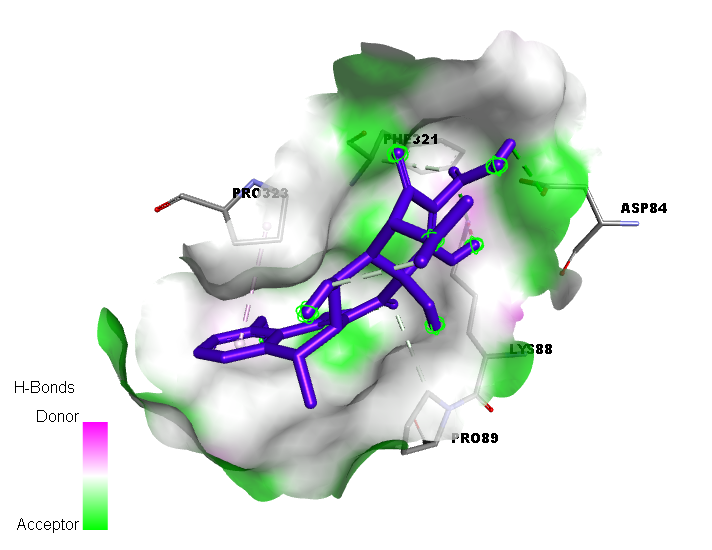** |
| GZMK | GZMK-WT-Bosutinib | **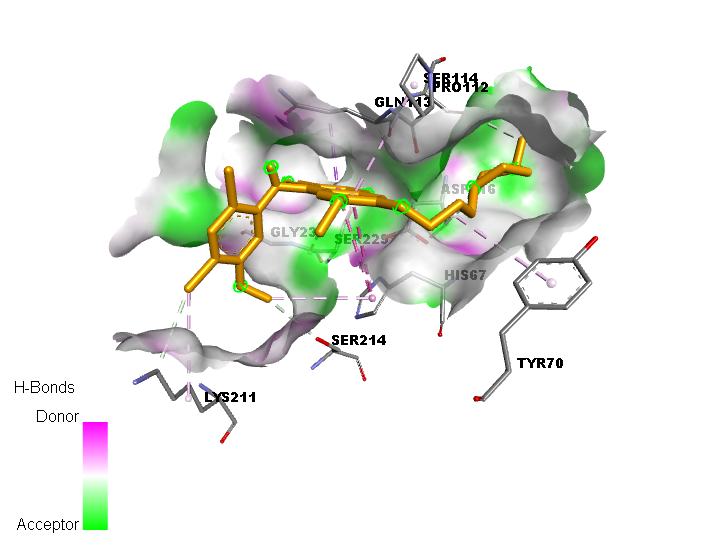** |
|  | A42P-Bosutinib | **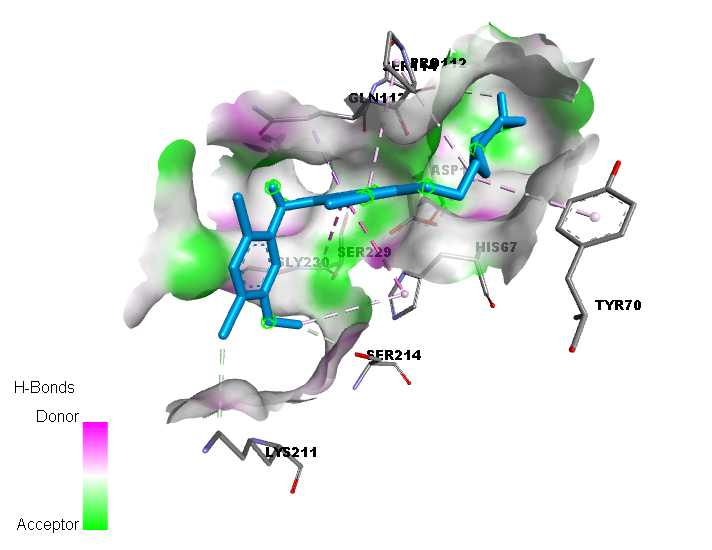** |
|  | L122P-Bosutinib | **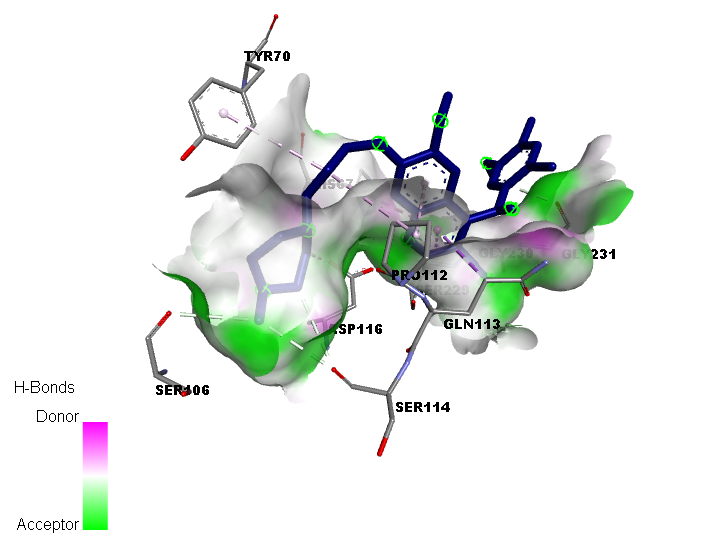** |
| OASL | OASL-WT-Astilbin | **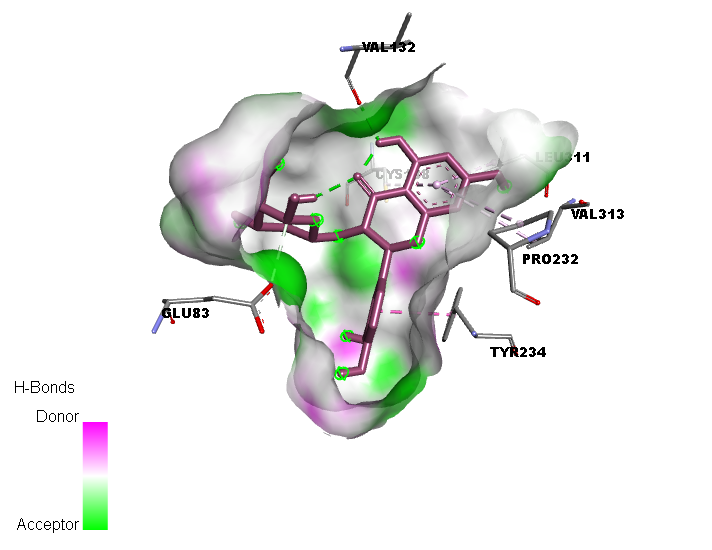** |
|  | **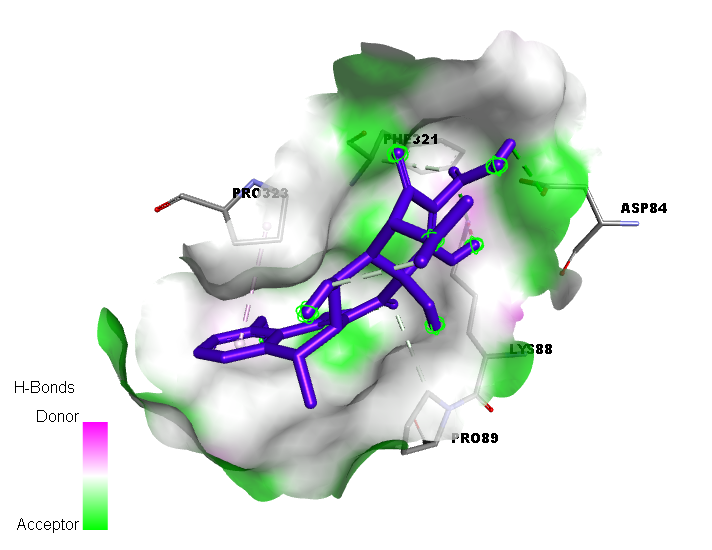**W216C-Astilbin |  |

**Supplementary Table 3.** MM/PBSA energy components (kcal/mol) of WT and mutant MMP8, GZMK, and OASL complexes

| Complex | Variant | ΔE_vdW | ΔE_ele | ΔG_polar | ΔG_nonpolar | ΔG_total |
| --- | --- | --- | --- | --- | --- | --- |
| MMP8 | WT | -37.50 | -115.76 | +143.13 | -4.25 | -14.38 |
|  | D253N | -13.50 | +118.95 | -108.35 | -1.60 | -4.51 |
|  | Y261S | -27.33 | -100.57 | +116.98 | -3.16 | -14.09 |
| GZMK | WT | -31.29 | +71.17 | -52.81 | -4.02 | -16.94 |
|  | A42P | -26.03 | +87.74 | -74.25 | -3.82 | -16.37 |
|  | L122P | -18.64 | +97.20 | -89.12 | -2.76 | -13.32 |
| OASL | WT | -35.22 | -14.42 | +40.04 | -3.80 | -13.40 |
|  | W216C | -37.25 | -44.63 | +66.55 | -4.29 | -19.63 |

| **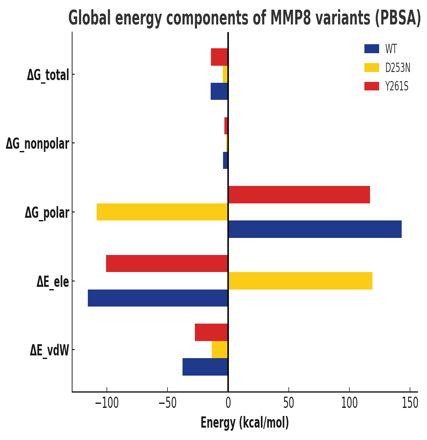** | **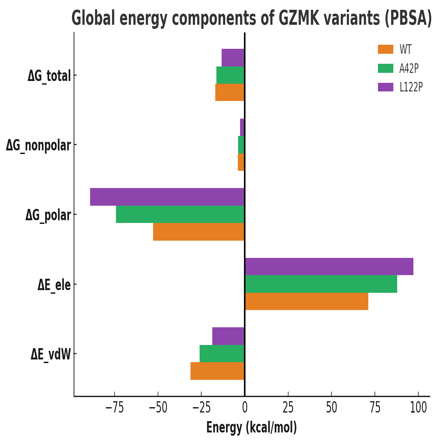** | **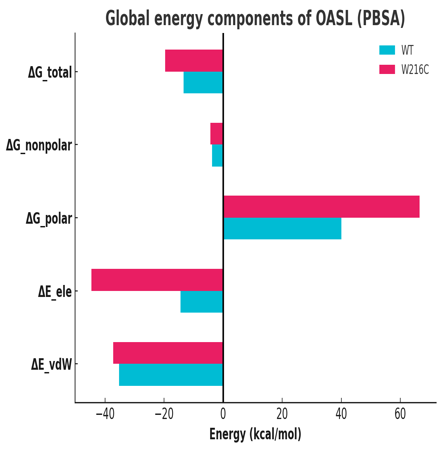** |
| --- | --- | --- |

**Supplementary Figure 4.** Decomposition of MM/PBSA binding free energy into van der Waals (ΔE_vdW), electrostatic (ΔE_ele), polar solvation (ΔG_polar), nonpolar solvation (ΔG_nonpolar), and total binding free energy (ΔG_total) for WT and mutant MMP8, GZMK, and OASL complexes. Energies are expressed in kcal/mol.
